# Roles of neuropathology-associated reactive astrocytes: a systematic review

**DOI:** 10.1186/s40478-023-01526-9

**Published:** 2023-03-13

**Authors:** Jill M. Lawrence, Kayla Schardien, Brian Wigdahl, Michael R. Nonnemacher

**Affiliations:** 1 Molecular and Cell Biology and Genetics Graduate Program, Drexel University College of Medicine, Philadelphia, PA USA; 2 Department of Microbiology and Immunology, Drexel University College of Medicine, Philadelphia, PA USA; 3 Center for Molecular Virology and Translational Neuroscience, Institute for Molecular Medicine and Infectious Disease, Drexel University College of Medicine, Philadelphia, PA USA; 4 Department of Neurobiology and Anatomy, Drexel University College of Medicine, Philadelphia, PA USA; 5 Sidney Kimmel Cancer Center, Thomas Jefferson University, Philadelphia, PA USA

**Keywords:** Neurotoxic reactive astrocytes, Activated astrocytes, A1 astrocytes, Neuroinflammation, Neurodegeneration

## Abstract

In the contexts of aging, injury, or neuroinflammation, activated microglia signaling with TNF-α, IL-1α, and C1q induces a neurotoxic astrocytic phenotype, classified as A1, A1-like, or neuroinflammatory reactive astrocytes. In contrast to typical astrocytes, which promote neuronal survival, support synapses, and maintain blood–brain barrier integrity, these reactive astrocytes downregulate supportive functions and begin to secrete neurotoxic factors, complement components like C3, and chemokines like CXCL10, which may facilitate recruitment of immune cells across the BBB into the CNS. The proportion of pro-inflammatory reactive astrocytes increases with age through associated microglia activation, and these pro-inflammatory reactive astrocytes are particularly abundant in neurodegenerative disorders. As the identification of astrocyte phenotypes progress, their molecular and cellular effects are characterized in a growing array of neuropathologies.

## Astrocyte biology

### History

The cellular environment of the central nervous system (CNS) is dominated by the ubiquitous presence of star-shaped glia cells. When first described by Rudolph Virchow in 1858, what he termed *neuroglia* were initially thought to be a form of connective tissue, the abundance of which simply served to hold neurons together like “glue” [2, 95]. In reality, the eponymous astrocytes perform a wide variety of critical functions that support neurons (cells that collectively comprise the circuitry responsible for brain function), synapses (physiological space between signaling neurons in which neurotransmission occurs), and the blood–brain barrier (a protective cellular layer that envelops blood vessels in the CNS), making them essential for maintaining CNS homeostasis.

### Astrocyte functions

Astrocytes promote survival of neurons and myelin-producing oligodendrocytes through the secretion of neurotrophic growth factors [2]. Synapse formation, pruning, and remodeling are all regulated by astrocyte functions, and established synapses continue to be strictly regulated by astrocytes. The broad topic of astrocyte biology and function is explored in a comprehensive review by Sofroniew and Vinters [113]. Briefly, to protect and support electrochemical neurotransmission, astrocytes extend processes that envelop the space between pre-synaptic and post-synaptic neurons, forming a tripartite synapse [2, 6, 113]. Rather than firing an action potential in response to excitation, astrocytes respond with a sharp increase in intracellular Ca^2+^ concentrations and the release of signaling gliotransmitters, such as glutamate and purines, or neuroactive steroids into the synaptic cleft to excite neurons, or to other astrocytes [113]. To prevent excitotoxicity, astrocytes assist with the termination of neuronal signaling via uptake of excess neurotransmitters from the synaptic space through densely clustered transporters expressed on astrocytic processes [40, 113]. Once taken up, the neurotransmitter is subsequently broken down and the inactive precursor components are released back into the synaptic cleft, where they are recycled by the neurons for future signaling [40].

As the most abundant cell type in the brain, astrocytes can form bidirectional contacts with both neurons and the microvascular endothelial cells lining cerebral blood vessels. When these associations are simultaneous, the astrocyte is able to transport oxygen, glucose, and other substrates from the blood to the neurons it supports [113]. Astrocytes can modulate local blood flow in response to neuronal activity by releasing vasodilators like nitric oxide or arachidonic acid, increasing blood flow and thus their provision of nutritional support for active neurons [79, 113].

Composed of a layer of microvascular endothelial cells, pericytes, and astrocytes, the blood–brain barrier (BBB) or neurovascular unit surrounds cerebral capillaries and protects the delicate CNS from the periphery while ensuring adequate blood supply [113]. The BBB strictly regulates what enters the brain parenchyma from the blood, with its low permeability maintained by tight junctions between the endothelial cells [78]. Its functional integrity relies on close associations of dense astrocyte processes, or endfeet, that ensheath blood vessel endothelial cells, forming a charged basement membrane, known as the glial limiting membrane [113, 120]. These processes are lined with various ion transporters, junctional proteins, and aquaporin-4 (AQP4), an astrocyte-specific water channel [113, 141]. Astrocytes also maintain integrity of outer and inner cerebrospinal fluid-brain barriers that similarly separate the brain parenchyma from the subarachnoid space and ventricles, respectively [15, 132]. A significant role of the BBB is restriction of peripheral immune cells from invading the CNS on a large scale [106]. Compromised barrier integrity can permit the infiltration of immune cells that release pro-inflammatory cytokines and toxic reactive oxygen species (ROS), leading to destructive neuroinflammation and potential neurodegeneration [106]. Thus, astrocyte function and support of the BBB is essential for protecting against neurotoxic insult.

### Astrocyte variations and reactivity

As opposed to being monolithic in function and morphology, astrocytes differ significantly depending on both tissue and cellular localization. Fibrous astrocytes have long fiber-like processes that associate with nodes of Ranvier along the axons that comprise the white matter, while protoplasmic astrocytes have highly branched processes that associate with neuronal somas and synapses that form the grey matter [2, 113, 120]. While these characterizations have been recognized for some time, functional distinctions have only recently been investigated. Historically, in response to damage, astrocytes have been characterized as adopting a reactive phenotype. In contrast to the typically quiescent state of mature astrocytes, reactive astrocytes can become highly proliferative, and this astrogliosis is the foundation for glial scar formation [5, 33, 75]. The presence of glial fibrillary acidic protein (GFAP), a cytoskeletal intermediate filament, has long been used as an astrocyte-specific marker, with increased GFAP in tissues of diseased or injured brains indicating cell proliferation and thus reactivity [75]. However, GFAP expression levels are known to be fluid and vary significantly from cell to cell, even in the absence of disease [12, 75]. Recently, more advanced and reliable measures of cell division have shown that astrocyte proliferation is not a universal response to all damage, but is instead quite limited in contexts of inflammation and neurodegeneration, regardless of increased GFAP expression [75]. In experiments utilizing proliferation markers, such as BrdU incorporation into newly-synthesized DNA and cell division-associated Ki67 staining, less than 3% of astrocytes responded with increased proliferation in an Alzheimer’s disease (AD) mouse model, and little to no proliferation was detected with treatment with lipopolysaccharide (LPS), an inflammatory signal [2, 54, 75, 142]. Further investigations involving genetic analysis of astrocytes under a variety of stimuli was shown to elicit distinct gene expression patterns [142]. To best support the CNS in a system that can suffer from a variety of insults, astrocytes have seemingly evolved a diverse reactive response. The work of Shane Liddelow and the late Ben Barres identified the genetic and functional changes that astrocytes undergo in response to different stimuli, and the mechanism by which these changes occur. In 2017, Liddelow and Barres reported their discovery of at least two distinct functional phenotypes that quiescent astrocytes can adopt, termed A1 and A2, laying the foundation for the current and evolving understanding of the role of astrocytes in disease [76].

Reactive phenotypic polarization depends on the nature of the inducing stimuli. Rather than eliciting a single response to CNS injury or insult, astrocyte reactivity is highly heterogenous. Borrowing from the nomenclature used to describe reactive macrophages and microglia, in response to tissue damage and ischemia, astrocytes adopt a neuroprotective A2 phenotype [75]. A2s fit the traditional reactive astrocyte profile and have proliferative functions, resulting in glial scar formation, debris clearance, and BBB repair [39, 142]. They upregulate neurotrophic factors and pro-synaptic thrombospondins, thereby promoting neuronal growth and supporting synaptic repair [19]. In contrast, neuroinflammation, infection, and aging induces a cytotoxic A1 reactive astrocyte phenotype [21, 75, 76]. Neurotoxic A1 reactive astrocytes are pro-inflammatory and associated with neurodegeneration and chronic neuropathic pain, in addition to a repression of functions related to supporting neuronal survival and synaptogenesis [72, 75, 76]. Additionally, inflammatory LPS treatment in aging mice further increased the proportion of hippocampal and striatal astrocytes expressing genes associated with the pro-inflammatory astrocytic phenotype and CXCL10 mRNA, indicating that aging and simultaneous inflammation have a cumulative effect on reactive astrocyte populations [21].

The use of A1/A2 nomenclature is not universally accepted, as such a stringent dichotomy fails to accurately represent the diversity within each subset of cells [30]. This system of classification can also give the false impression of reactive states being either entirely “helpful” or “harmful”, when in reality these reactive states likely evolved to serve various functional purposes [30]. It is more accurate to describe astrocyte reactive states in terms of molecular expression patterns and functional changes, as described in detail in a consensus statement published by Escartin et al. [30]. However, as the A1/A2 nomenclature is still used in a large number of reports, we have attempted to use language consistent with those used by the authors being referenced. As such, the terms A1, A1-like, neuroinflammatory, neurotoxic, or pro-inflammatory describe the pathology-associated reactive astrocyte phenotype, and the terms A2, A2-like, and neuroprotective, or proliferative describe the repair-associated reactive astrocyte phenotype.

Astrocyte responses to infection, injury, and disease are highly significant due to the critical roles they possess. This manuscript will provide a detailed overview of the etiological contributions of the recently-characterized pro-inflammatory reactive astrocyte phenotype in the context of various neuropathologies [21].

## Activation and polarization of astrocytes

### Microglia activation

The induction of polarization of a quiescent astrocyte to a pro-inflammatory phenotype is mediated by another widespread glial cell population, microglia, which serve as the resident macrophages of the CNS. Microglia and astrocytes interact frequently in a combined effort to support CNS function. In the neurovascular unit at the cerebral blood vessels, microglia are dispersed throughout the tightly clustered astrocytes and endothelial cells, allowing them to detect insults entering from the periphery [78]. When neurons undergo apoptosis, to prevent the dying cell from influencing local neurons, astrocytes and microglia form a microscar around it. Astrocytic projections surround and perforate the neuronal soma, allowing the microglia to more easily phagocytose [67]. Due to the nature of their relationship, there is a high degree of crosstalk between astrocytes and microglia. As the more mobile microglia come closer in proximity with an astrocyte, their projections which are coated with Integrin-b1 mechanosensors, branch out to contact the astrocyte projections [67, 68]. At the site of microglial-astrocyte projection contact, upregulation of the microglial projection Integrin-b1, a membrane receptor that detects and responds to mechanical stimulation, likely facilitates cytoskeletal remodeling of microglial branches, thus improving their migratory and phagocytic functions [67, 68]. These close associations also allow for efficient paracrine signaling from microglia to astrocytes.

In a normal environment, the microglia patrol local tissue environment for damage or infection [78]. In response to their environment, microglia polarize into the pro-inflammatory and anti-inflammatory phenotypes, with intermediate stages indicating phenotypical changes are more fluid rather than dichotomous [67, 78]. A variety of stimuli, including damage, neurodegenerative disease, chronic or acute inflammation, and infection, can activate microglia and result in an innate immune response [78]. This activation occurs typically via the cell surface receptor, Toll-like receptor 4 (TLR4) and in response to both pathogen-associated molecular patterns (PAMPs) and damage-associated molecular patterns (DAMPs) [78]. Microglial activation can be sustained via a self-feedback loop involving the high-mobility group protein box-1 (HMGB1), a DNA-binding protein expressed by activated microglia and macrophages to assist with transcription of genes associated with a pro-inflammatory response [56, 78]. As rising concentrations of HMGB1 are released by activated microglia, secreted HMGB1 can then go on to interact with surface TLR4, propagating further microglia [78]. HMGB1 has been associated with promoting neurodegeneration, synaptic depression, and contributing to cognitive dysfunction, a topic thoroughly covered by Paudel et al. [24, 97]. In a murine dementia model, HMGB1 complexed with CXCL12 reduced BBB integrity and promoted immune cell infiltration, resulting in increased neuroinflammation, particularly in the hippocampus [24]. CXCR7 inhibits the complex, in part by sequestering CXCL12, thereby preventing HMGB1-induced neuroinflammation, regulating appropriate reactive microglia and astrocyte proportions, maintaining BBB integrity and promoting overall CNS homeostasis [24].

**Fig. 1:**
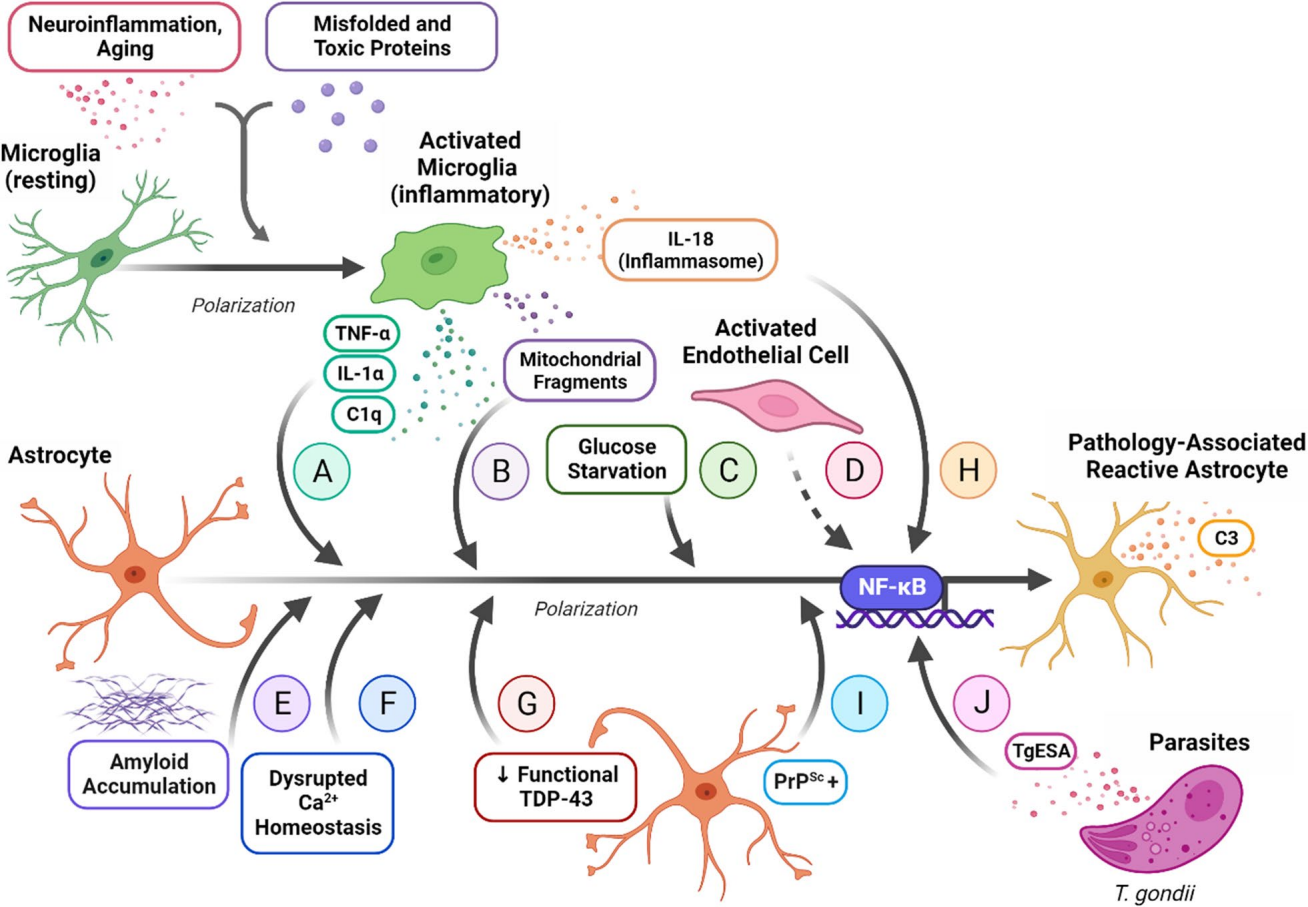
Mechanisms of neurotoxic A1-like (C3+) reactive astrocyte polarization. **a** Neuroinflammation and the presence of misfolded, cytotoxic, or pathological proteins activates microglia, inducing an M1 phenotype that secretes TNF-α, IL-1α, and C1q [66, 76]. This combination of inflammatory signals is the primary mechanism of inducing neurotoxic astrocyte reactivity. **b** Microglial activation in response to pathogenic proteins is associated with excessive Drp1-Fis1-binding-induced mitochondrial fission, and subsequent release of mitochondrial fragments, a process that may work in conjunction with TNF-α, IL-1α, and C1q signaling to efficiently induce neuroinflammatory phenotype polarization [52]. **c** Prolonged glucose starvation induces astrocyte polarization to a pro-inflammatory phenotype that specifically displays significant upregulation of unfolded protein response genes [62]. **d** Activated endothelial cells induce astrocytic upregulation of C3, the extracellular matrix remodeling protein Decorin, and phagocytic functions [122]. **e** AD-associated amyloid accumulation is associated with activation of the NF-κB pathway in astrocytes, which adopt a C3 + neurotoxic phenotype [70, 74]. **f** In a TMT-intoxication model of AD, an influx of Ca^2+^ enters astrocytes via voltage-gated ion channels, resulting in mitochondrial membrane depolarization, upregulation of ROS and NOS, and pro-inflammatory astrocyte-associated C3 expression [28]. **g** Astrocytes with ALS-associated loss of functional TDP-43 adopt a neuroinflammatory phenotype, a response that is correlated with a loss of oligodendrocytes and indirect motor neuron damage [100]. **h** Exposure to IL-18 processed by the activated microglial NLRP3 inflammasome induces a C3 + synaptotoxic astrocyte response [44]. **i** Prion-propagating PrP^Sc^ + astrocytes upregulate C3 but also undergo distinct transcriptional changes that are considered pan-reactive [38]. **j** Astrocytes exposed to the *T. gondii* excreted-secreted antigens (TgESAs) underwent polarization to a C3 + reactive phenotype via NF-κB pathway activation [51]

**Fig. 2:**
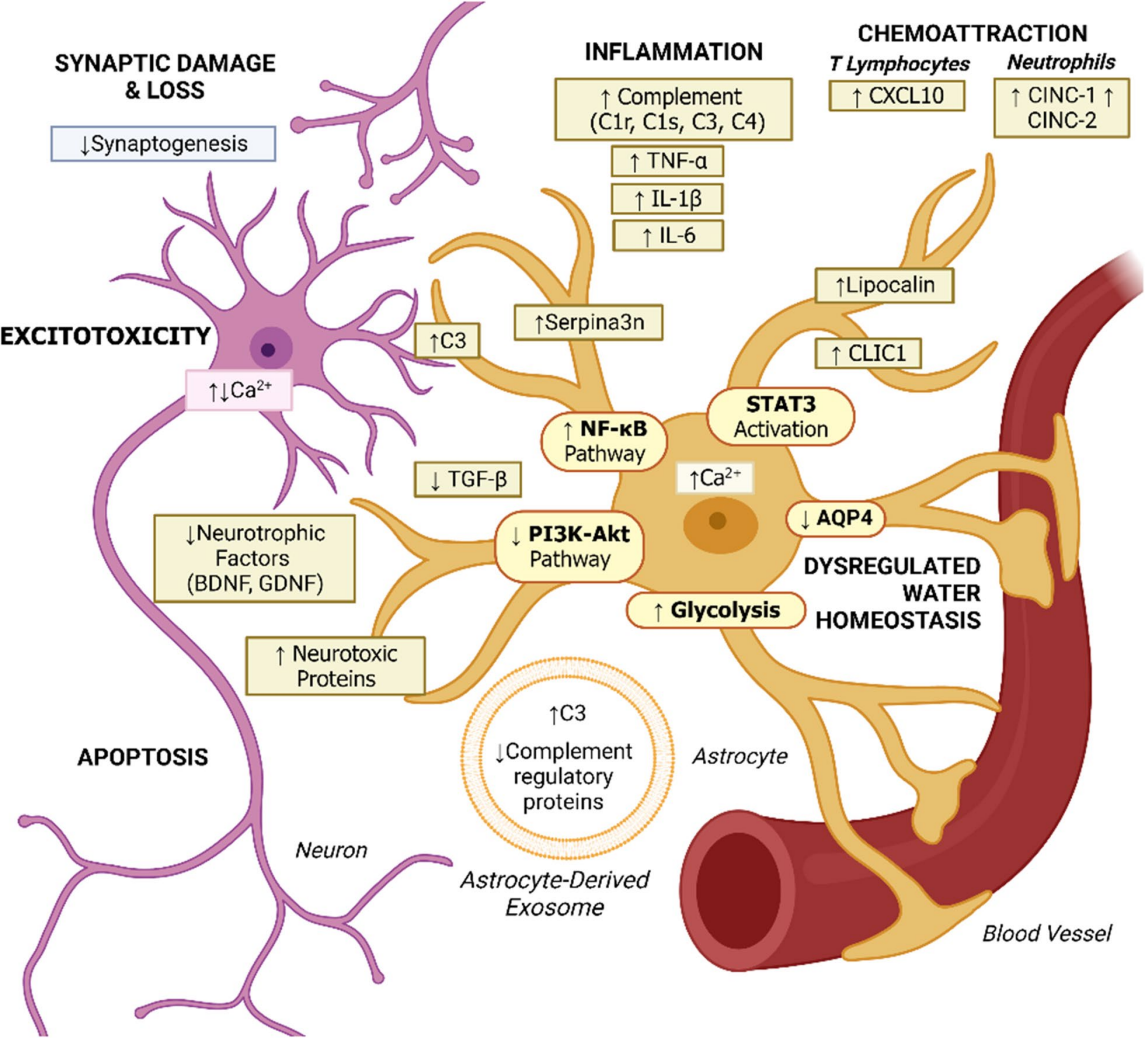
Molecular and cellular effects of A1-like C3 + astrocytes. Neurotoxic reactive astrocytes upregulation of the pro-inflammatory NF-κB signaling pathway results in increased complement component expression (C1r, C1s, C3, C4) and synaptic damage [43, 74, 75, 117, 142]. Other pro-inflammatory mediators expressed by A1-like astrocytes include IL-1β, TNF-α, and IL-6 [63]. Hyperphosphorylation and activation of the STAT pathway has been reported in inflammatory reactive astrocytes associated with injury response [144]. Inhibition of the anti-inflammatory PI3K-Akt pathway results in reduced TGF-β expression, and downregulation of synaptogenic factors and neurotrophic signals, including BDNF and GDNF, contribute to synapse loss and apoptosis [129, 135]. Increased glycolytic functions and secretion of astrocyte-derived exosomes containing increased concentrations of C3 have been identified in neuropathology-associated reactive astrocytes, specifically in the context of AD [34, 145]. Changes in expression of AQP4, resulting in dysregulated water homeostasis at the BBB, are another hallmark of A1-like astrocytes [60]. Other factors secreted by A1-like neurotoxic reactive astrocytes with unidentified functions include Serpina3n, Lipocalin, and CLIC1, with several neurotoxic factors yet to be identified [83, 129]. Chemoattractive signals for T lymphocytes, such as CXCL10, and neutrophils, such as CINC-1 and CINC-2, are also produced by neurotoxic astrocytes, potentially contributing to immune cell infiltration and establishing a pro-inflammatory environment [21, 41, 57, 75, 121]. Secreted C3 binds to the C3aR receptor on local neurons, resulting in subsequent dysregulation of intraneuronal Ca^2+^ homeostasis and excitotoxicity [74]

In a neuroinflammatory or neurodegenerative context, activated microglia will adopt an amoeboid morphology, perform phagocytic functions, produce ROS, and secrete an array of pro-inflammatory mediators, among which are tumor necrosis factor-α (TNF-α), interleukin-1α (IL-1α), and complement component subunit 1q (C1q) [76, 78]. It is the exposure to these three specific signals that triggers a series of downstream intracellular effects that alter the transcriptional activity of a local non-reactive astrocyte, converting it to an neurotoxic phenotype [75, 76] (Fig. 1a). The NF-κB signaling pathway upregulates a wide array of inflammatory response genes, as explored by Pahl et al. [93], including complement component 3 (C3), and likely plays a critical role in astrocyte polarization (Figs. 1a, 2, Table 1) [75, 86, 93]. When this pro-inflammatory pathway is activated by ligand binding to cytokine receptors, intracellular IκBα is phosphorylated, promoting it to dissociate from a complex with p50 and p65 [135]. IκBα is subsequently degraded, while p50 and p65 are phosphorylated, permitting their translocation from the cytosol to the nucleus where they can influence gene expression [135]. When astrocytes were treated with activated microglia-conditioned media (MCM), increased levels of phosphorylated p65 and nuclear p65 were detected, suggesting the activation of the NF-κB pathway [135]. Furthermore, treatment with the NF-κB inhibitor PDTC prevents reactive astrocyte polarization [135]. In the absence of microglia, astrocyte monocultures exposed to LPS secrete negligible levels of ROS, TNF-α, and nitric oxide (NO) and remain inactivated, highlighting the necessity of astrocyte and microglia crosstalk to create a sufficient astrocyte-mediated inflammatory response [20, 78].

**Table 1:** Markers used to identify astrocyte phenotypes

Protein	Function	References
*Non-specific astrocyte markers*		
GFAP	Cytoskeletal intermediate filament	Liddelow and Barres [75], Boulay et al. [12], Liddelow et al. [76]
S100β	Ca^2+^-binding protein	Liddelow et al. [76]
ALDH1L1	Aldehyde dehydrogenase	Serrano-Pozo et al. [108]
*Neuroprotective reactive astrocyte markers*		
S100A10	Ca^2+^-binding protein	King et al. [58]
PSD95	Post-synaptic density protein	Singh et al. [112]
TGF-β	Growth factor	Xu et al. [135]
COX2	Cyclooxygenase	Hensel et al. [42]
GDNF	Neurotrophic factor	Wang et al. [129]
BDNF	Neurotrophic factor	Wang et al. [129]
*Pro-inflammatory reactive astrocyte markers*		
C3	Complement component	Liddelow and Barres [75], Zamanian et al. [142], Wang et al. [129], Hou et al. [44]
C1r, C1s, C4	Complement components	Liddelow and Barres [75], Zamanian et al. [142]
Serpina3n	Peptidase inhibitor	Clarke et al. [21], Masvekar et al. [83], Singh et al. [112]
Serping1	Serine protease inhibitor	Fang et al. [31], Kim and Son [57]
ROS	Reactive oxygen species	Joshi et al. [52], Dragić et al. [28]
STAT3	Transcription factor	Zhang et al. [144]
TNF-α	Cytokine	Joshi et al. [52]
p50, p65	NF-κB Pathway	Xu et al. [135]
SRGN	Proteoglycan	Klemens et al. [60]
Lipocalin 2	Iron-sequestration	Wang et al. [129]
GBP2	Guanylate-binding protein	Ugalde et al. [126]
ACSL5	Acyl-CoA synthetase	Klemens et al. [60]
CLIC1	Cl^−^ channel	Masvekar et al. [83]
CXCL10	T cell chemoattractant	Clarke et al. [21], Kim and Son [57]
CINC-1, CINC-2	Neutrophil chemoattractant	Clarke et al. [21], Kim and Son [57]

### Alternative neuroinflammatory reactive astrocyte induction pathways

#### Mitochondrial fragmentation

The pro-inflammatory cocktail of TNF-α, IL-1α, and C1q may not be the only mechanism required for the induction of a neuroinflammatory astrocytic phenotype. A pathological feature of many neurodegenerative disorders is the presence of extracellular dysfunctional mitochondrial fragments that appear to propagate the inflammatory response in microglia [52]. In contrast to normal mitochondrial fission, dysfunctional mitochondrial fragmentation occurs when there is excessive binding of activated Dynamin-related protein 1 (Drp1) to the mitochondrial fission 1 (Fis1) receptor, and results in the release of these fragments into the extracellular space [52]. Further research indicates that exposure to neurotoxic proteins released in the context of neurodegenerative diseases, including amyloid-β in AD, activates microglia [70]. This process is associated with Drp1-Fis1-induced mitochondrial fragmentation, as evidenced by the loss of microglia activation when treated with P110, a pharmacological inhibitor of excessive mitochondrial fission [52]. When primary murine astrocytes were exposed to MCM collected from microglial models of Huntington’s disease (HD) and amyotrophic lateral sclerosis (ALS), they polarized to an A1-like phenotype and increased secretion of TNF-α and IL-1β [52] (Fig. 1b). In addition, these astrocytes also displayed signs of their own mitochondrial fragmentation and dysfunction, with decreased ATP production and increased cytotoxic ROS production [52]. Interestingly, astrocyte activation using TNF-α, IL-1α, and C1q can be inhibited via P110 treatment, suggesting that Drp1-Fis1-induced mitochondrial fragmentation contributes to A1-like polarization [52]. Additionally, treatment of astrocytes with LPS resulted in increased expression of an ATP-sensitive K^+^ channel subunit, Kir6.2, that mediates Drp1-induced mitochondrial fission [114]. In summary, extracellular dysfunctional fragmented mitochondria released by activated microglia induces polarization of local astrocytes, releasing their own mitochondrial fragments, and propagating neurodegenerative injury.

#### Nutrient starvation

It is unlikely that neurotoxic polarization is strictly limited to contexts of inflammation or injury; rather, it may also be induced by other environmental factors. Using rat cerebral cortical astrocytes, an in vitro model of anorexia nervosa or long-term undernutrition was induced by starving the cells of glucose [62]. At 15 days, the gene expression profiles of the astrocytes had shifted to match that of an A1-like phenotype, with unfolded protein response genes being particularly upregulated in a pattern consistent with other metabolic disorders [62] (Fig. 1c). This suggests that chronic undernutrition can elicit an astrocyte-mediated neuroinflammatory response, although the exact underlying mechanisms remain unknown [62].

#### Endothelial cell activation

Recent evidence indicates possible transcriptional variation within the neurotoxic reactive astrocyte phenotype, resulting in a cell type-dependent polarization. When conditioned media from LPS-activated endothelial cells was applied to astrocytes, immunostaining of primary mouse cells revealed a substantial 86% increase in astrocytic expression of C3, a functional marker of pro-inflammatory A1-like polarization, compared to control [122]. Notably, microglia-conditioned media only elicited a 44% increase in C3 expression, suggesting that activated endothelial cells may be another source of inflammatory astrocyte polarization [122] (Fig. 1d). RNA-seq analysis revealed that the expression profile of astrocytes activated by endothelial cells was not identical to those of astrocytes activated by microglia. Specifically, this reactive subset upregulated expression of genes associated with extracellular matrix remodeling, such as Decorin [122]. Functionally, C3^+^ Decorin^+^ double-positive reactive astrocytes activated by endothelial cell signaling retained their phagocytic responses, while the C3^+^ Decorin^−^ pro-inflammatory reactive astrocytes induced by microglial signaling lose this function [122] (Fig. 1d). This may have important implications for tissue-specific neurotoxic astrocyte responses, particularly at CNS barriers.

### Regulation of astrocyte polarization phenotype

#### Inflammatory pathways

Given the degree of cross-talk between astrocytes and microglia, it is not surprising that their influence on each other can be bi-directional. In a study modeling AD using amyloid-β 42 (Aβ42)-activated MCM, treatment with the MCM resulted in the expected astrocyte polarization, as evidenced by increasing levels of inflammatory A1-associated C3 and decreasing levels of A2-like-associated transforming growth factor β (TGF-β) [135] (Fig. 2). Direct treatment of astrocytes with Aβ42 was unable to elicit these responses alone, emphasizing the importance of microglia facilitation of astrocyte activation [135]. This response was suppressed by pre-exposing astrocytes to milk fat globule epidermal growth factor 8 (MFG‐E8), endogenously expressed by both microglia and astrocytes [135]. Serving as a pro-inflammatory astrocyte blockade, MFG-E8 specifically downregulated NF-κB signaling and upregulated the PI3K-Akt pathway, highlighting the important role they play in astrocyte polarization [135]. In contrast to the pro-inflammatory NF-κB signaling pathway, secretion of TGF-β downstream of the PI3K-Akt pathway serves as an anti-inflammatory signal [135]. In an in vitro study examining cell activation in mixed glial cell cultures, microglia treated with the inflammatory cytokine granulocyte–macrophage colony-stimulating factor (GM-CSF) were polarized to an activated phenotype and began to proliferate rapidly [57]. In contrast, in the presence of A2-like astrocytes, microglia proliferation similarly increased, but an anti-inflammatory phenotype was induced [57].

In addition to microglia-astrocytic crosstalk, there is also a degree of signaling between astrocytes and macrophages. Anti-inflammatory macrophage phenotype-conditioned media (achieved via IL-4 stimulation) actively suppresses polarization to an inflammatory phenotype in a process facilitated by the long non-coding RNA signaling axis, NEAT1/miR-224-5p/IL-33 that regulates macrophage activation [77]. In some pathological contexts in which tissue repair is of primary importance, simultaneous promotion of neuroprotective and inhibition of neuroinflammatory astrocyte phenotypes can occur. Damaged dopaminergic neurons can secrete a chemokine-like signal, prokineticin-2 (PK2), which can go on to bind to PK2 receptor 1 (PKR1) expressed by astrocytes [91]. This induces a shift in gene expression to that of an A2-like phenotype, stimulates proliferation and astrocyte chemotaxis to the site of damage, and upregulates both glycolytic and aerobic ATP production pathways [91]. PK2-PKR1 binding also downregulates pro-inflammatory cytokines and pro-oxidative factors associated with an A1-like phenotype [91].

#### Circadian rhythm

Metabolism and inflammation in both the CNS and periphery are also regulated by circadian rhythms, established in the hypothalamic suprachiasmatic nucleus (SCN) [69]. Cells throughout the body are synchronized to the environmental light–dark cycles, forming a cyclic pattern of molecular actions respective to cell type over the course of a day and night. This innate molecular clock encompasses a series of transcriptional-translational feedback loops, with both a positive and negative limb. The positive progression involves BMAL1 transcription factor heterodimers and CLOCK or NPAS2 proteins, while the negative feedback loop involves inhibiting BMAL1 via increased levels of its own transcriptional targets: PER, CRY, and REV-ERB [69]. In neurodegenerative disorders, circadian clock protein gene expression and circadian rhythm can become dysregulated [69]. Deletion of BMAL1 in a mouse model results in not just the absence of a synchronized molecular clock, but also induces significant astrogliosis and subsequent neuroinflammation and synaptic damage, suggesting a regulatory role for BMAL1 in astrocyte reactivity [69, 90]. Indeed, astrocytes lacking BMAL1 spontaneously polarize to an A1-like phenotype [69]. Further investigation of this cell-autonomous regulatory mechanism revealed that BMAL1 expression inhibits pro-inflammatory astrocyte responses via depression of glutathione-S-transferase signals [69].

## Characteristics of neuroinflammatory astrocyte function

### Molecular and cellular effects

#### Inflammatory factors

The neurotoxic effect of reactive astrocytes is multi-faceted and reflects the various supportive roles astrocytes serve. Due to their close association with neurons, reactive astrocytes can have significant deleterious effects on neuronal survival via downregulation of neurotrophic signals and secretion of neurotoxic factors [75, 76, 142]. Pro-inflammatory astrocytes facilitate synaptic damage by downregulating synaptogenic signals while upregulating complement cascade genes such as *C1r*, *C1s*, *C3*, and *C4* that result in synaptic damage and reduced connectivity between neurons [43, 74, 75, 117, 142] (Fig. 2). The NF-κB signaling pathway also appears to be active in neurotoxic reactive astrocytes, contributing to C3 production and release [74, 135] (Fig. 2). In addition to secreting inflammatory complement components like C3, pro-inflammatory reactive astrocytes also produce chemoattractants, such as cytokine-induced neutrophil chemoattractants 1 and 2 (CINC-1 and CINC-2), as well as CXCL10, a chemoattractive signal for T lymphocytes, which may encourage immune cell infiltration and contribute to establishment of a pro-inflammatory CNS environment [21, 41, 57, 75, 121] (Fig. 2).

#### AQP4

A1-like neurotoxic astrocytes also display modified expression of astrocyte-specific proteins associated with CNS barrier function such as AQP4 [60] (Fig. 2). In the context of canine demyelinating disease, the presence of C3 + astrocytes was associated with a progressive loss of AQP4 expression [60] (Fig. 2). AQP4 is an aquaporin specific to astrocyte projections, the loss of which is a marker of barrier dysfunction in other neuropathologies and may contribute to overall disruption of CNS homeostasis [11, 60]. On astrocytes that support the BBB, this channel specifically plays an important role in providing the CNS with water and maintaining local osmolarity. In acute cerebral ischemia or water intoxication, the movement of water facilitated by AQP4 results in cytotoxic brain edema; therefore, in this context reduced AQP4 expression can be beneficial [81, 141]. In contrast, in persistent ischemia or brain tumor, vasogenic edema arises from iso-osmolar fluid leaking in through a damaged BBB. In this case, AQP4 counteracts these effects by reabsorbing excess fluid, and the loss of AQP4 significantly exacerbates the severity of the edema [94, 141]. Another study using a mouse model with an AQP4 deletion, reported both an increased threshold for seizure induction as well as an increased seizure duration. l [11]. This seemingly contradictory relationship can be attributed to the multi-dimensional role AQP4 serves on astrocytes that support synapses. In the hypo-osmotic context of hyponatremia, the intracellular concentration of Na^+^ is higher compared to the extracellular concentration within the synaptic space [11, 89]. This concentration gradient drives water through AQP4 channels into the astrocyte. As the astrocyte swells, the volume of the synaptic space decreases, thereby increasing the relative local neurotransmitter concentration and increasing neuronal excitability and reducing the threshold for seizure to occur [11, 89]. In the absence of AQP4 and astrocyte swelling, the synaptic volume would remain the same and neuronal hyperexcitability would not occur, thus increasing seizure threshold [11]. However, AQP4 is also associated with K^+^ clearance kinetics. K^+^ is released by neurons through voltage-gated potassium channels into the synapse to hyperpolarize the membrane and ultimately terminate firing. Astrocyte clearance of synaptic K^+^ helps to maintain the low extracellular concentrations necessary for K^+^ ion flow to occur. In the absence of AQP4, the reduction of extracellular K^+^ is significantly delayed, prolonging depolarization and thus seizure duration [11]. Overall, AQP4 appears to support normal signal termination, and its loss disrupts this function.

#### Ca^2+^ signaling

Rather than passively contributing to neuropathology by retraction of vital functions, there is a great deal of evidence to suggest that neurotoxic reactive astrocytes actively influence disease progression through mechanisms such as altered Ca^2+^ signaling. In pharmacologically-induced seizures, pro-convulsive Ca^2+^ signaling was shown to increase in astrocytes, possibly contributing to neuronal loss [26, 109]. Astrocytes that were most closely localized to amyloid plaques within a mouse model of AD demonstrated the greatest Ca^2+^ signaling, with similar spatiotemporal patterns being observed in models of ischemic stroke [27, 65, 109]. Increased Ca^2+^ signaling from reactive astrocytes may also influence gliotransmission, inhibit synaptic remodeling, and result in damage to neurons [109] (Fig. 2). Conversely, astrocytes in a rodent model of HD displayed reduced Ca^2+^ signaling, which was also observed to have corresponding behavioral ramifications, such as increased repetitive self-grooming [109, 139]. Changes in Ca^2+^ signaling frequency and amplitude in reactive astrocytes may occur via receptor-mediated Ca^2+^ signaling, transmembrane Ca^2+^ pathways, or through mitochondrial Ca^2+^ release. These underlying mechanisms and their respective consequences vary across different neuropathologies and disease states, a topic which is more thoroughly-explored in Shigetomi et al. [109].

### Neuropsychological effects

#### Stress responses and depressive behaviors

With the wide array of molecular and cellular effects neurotoxic reactive astrocytes exert, higher level neurological functioning is impacted. In mice with deficient expression of the anti-inflammatory cytokine IL-10, treatment with LPS escalates the pro-inflammatory response of the already higher proportion of A1-like astrocytes [144]. Neurotoxic astrocyte responses are associated with hyperphosphorylation and activation of signal transducer and activator of transcription 3 (STAT3), a transcription factor associated with damage responses, and demonstrate greater phagocytic capabilities (Fig. 2). Behavioral analysis of the mice revealed depressive behaviors and significantly impaired learning and memory function, reversible by treatment with the absent IL-10 [144]. In a mouse model of chronic social defeat stress (CSDS), reduced neuronal firing rates associated with displaying depression-like symptoms was observed [37]. Astrocytes harvested from the medial prefrontal cortex (mPFC) and hippocampus of stressed mice and were found to have adopted A1-like phenotypes [37]. Subsequent to astrocytic suppression via stereotaxic injection, the depressive behaviors and dampened neuronal activity elicited by the A1-like phenotype were reversed, thus indicating a role for astrocyte polarization and depressive behaviors in chronic psychological stress [37]. In mice with depleted microglial populations, astrocytes failed to polarize to an A1-like phenotype in response to chronic stress, thereby successfully preventing signaling impairment and depressive behaviors. This phenomenon suggests that the previously described process requires microglia to first respond to the chronically stressed environment to induce the activation of astrocytes [37]. In a chronic mild stress (CMS) mouse model of major depressive disorder (MDD), collected hippocampal tissue displayed decreased GFAP and branch density, increased neuroinflammatory phenotype-associated transcripts detected by RT-qPCR, and increased C3 and Serping1 protein expression detected by western immunoblot analysis, suggesting that neurotoxic reactive astrocyte activation may be present in MDD [31]. This response does not appear to be limited to mammals; analysis using a zebrafish model of delayed stress responses and post-traumatic stress disorder (PTSD) identified a significant increase in the proportion of A1-like astrocytes in the astrocyte population [137, 138]. Markers of chronic inflammation, including IL-1β and TNF-α, can be detected in conditions characterized by hypothalamic–pituitary–adrenal (HPA) axis dysregulation, like PTSD, with higher levels being associated with symptom severity [96, 137]. The results obtained from zebrafish may indicate that this chronic inflammation can induce an on-going neurotoxic astrocyte response, potentially contributing to some neurological and cognitive symptoms [137].

#### Cognitive impairment

Post-operative cognitive dysfunction (POCD), a condition that may arise following the administration of general anesthesia, particularly in aging populations, appears to be in part due to a microglia-mediated A1-like astrocyte response [71]. The synaptic inhibition that underlies the effect of general anesthesia is associated with increased levels of the inhibitory neurotransmitter GABA in the synaptic cleft, for which astrocytes express receptors, which results in temporarily repressed ligand-gated ion channels [71]. When the transient inhibitory currents fail to subside after the removal of anesthesia, cognitive deficits can result. This can be modeled in mice using etomidate injections. Microglia activation is increased shortly after treatment, and gradually hippocampal A1-like astrocyte levels rise over the next few weeks, resulting in poorer cognitive outcomes [71]. This suggests that in some susceptible or vulnerable individuals, general anesthesia elicits chronic microglia and neurotoxic astrocyte activation, and that this response can be linked to cognitive impairment [71].

In summary, unlike typical astrocytes that promote neuronal survival, support synapses, and maintain brain-barrier integrity, A1-like neurotoxic astrocytes downregulate supportive functions and alter the expression of proteins critical to normal astrocyte function, like AQP4 (Fig. 2). A1-like astrocytes also begin to secrete neurotoxic factors, complement components, like C3, and chemokines, like CXCL10, that may recruit T cells across a compromised BBB into the CNS [75] (Fig. 2). The abundance of pro-inflammatory A1-like astrocytes is associated with neuropathic pain, several chronic neurodegenerative disorders, as well as normal aging [8, 21, 72, 75, 76].

### Role in normal aging

The environment of the aging brain can exacerbate inflammatory effects and contribute to gradual neuronal damage. During the course of normal aging, as opposed to age-associated pathologies like AD, glia cells undergo a variety of physiological and functional changes. As a consequence of diminished cytoskeletal remodeling capabilities, microglia may begin to display reduced branches, decreased Integrin-b1 expression, and their populations can become more irregularly distributed [67, 68]. In addition to promoting neuroprotective signaling pathways, microglia in an aging brain upregulate expression of immune system response receptors, effectively becoming more sensitive to insults, and increasing production of pro-inflammatory signals, including TNF-α, IL-1α, and C1q [67].

Astrocytes in aging murine brains have been shown to upregulate Ca^2+^ signaling to the point of negatively influencing neuronal signaling [109]. The proportion of A1-like astrocytes increases with normal aging, due to associated microglia activation and signaling with TNF-α, IL-1α, and C1q that induces polarization to a pro-inflammatory phenotype [21, 75, 76]. This process may underly age-associated cognitive deficits and increased vulnerability to injury [21]. For example, the risk of developing a prolonged or permanent disability from a traumatic brain injury (TBI) is significantly higher in elderly populations [29]. In a mouse model of TBI recovery, the effect on astrocytes was greatest in the oldest mice, specifically a loss of AQP4 expression [29]. While the astrocytes in this study did not clearly conform to either an A1-like or A2-like phenotype, but rather showed characteristics of both, the dysregulation of AQP4 and upregulation of complement and inflammatory pathways are hallmarks of the neurotoxic reactive phenotype [29].

Astrocyte phenotypic changes during aging have been analyzed across different brain regions, typically through the use of murine models. Among mouse hippocampal and striatal astrocytes, normal aging induced A1-like polarization among 2 year-old mice compared to 10 week-old mice, as evidenced by increased levels of A1-like astrocyte-associated transcripts, including Serpina3n, C4B, C3, and CXCL10 [21] (Fig. 2). In a meta-analysis of transcriptomic data collected from human prefrontal cortex (PFC) samples, individuals in the 58–80 years-old range had increased levels of pan-reactive astrocyte markers, when compared to younger individuals [98]. This shift in the astrocyte population from largely quiescent to one with greater proportions of both neuroinflammatory and neuroprotective reactive astroctyes in the PFC may play an important role in the normal cognitive decline observed with aging [98]. Other studies looking at aging murine dopaminergic neurons observed a more distinct change, with a reduction in the level of A2-like markers, brain-derived neurotrophic factor (BDNF) and glial cell line-derived neurotrophic factor (GDNF), and a corresponding increase in pro-inflammatory A1 markers, C3 and lipocalin-2 (Lcn2) [129] (Fig. 2, Table 1). This may suggest that the changes in reactive astrocyte populations may differ depending on localization, with PCF-resident astrocytes responding differently compared to those in the midbrain. Whether there is a chronological pattern to this development, such as a subset of astrocytes becoming polarized due to microglial activation, followed by other astrocytes becoming polarized to a neuroprotective phenotype in response to reactive astrocyte-induced damaged, is currently unclear.

The hypothalamus is particularly important in aging, as the accumulation of “exhausted” scenescent glial cells in this region is thought to contribute to some of the cognitve and emotional regulation changes that occur during normal aging [119]. In a transcriptional analysis of hypothalamic glial cells in aging mice, it was found that these microglia had increased expression of programmed cell death 1 (PD-1) marker and the pro-inflammatory astrocyte phenotype-inducing factors TNF-α, IL-1α, and C1q [119]. This suggests that with aging, the surveying microglia evoke an inflammatory response, but also become exhausted as they do so. The astrocytes also expressed A1-like transcripts, as well as p16^INK4a^, a marker of scenescence, indicating a similar “exhaustion” response as the microglia [119]. These changes correlated with observed behavioral dysfunction and motor discordination, likely attributable to disruption of the HPA axis [119].

The neuroinflammatory astrocyte response in the brain that arises in advanced age is compounded by inflammation. In the absence of activated microglia cytokine secretion, age-induced astrocyte reactivity is reduced, supporting the role of activated microglia in age-associated A1-like responses [21]. In an aging mouse model treated with LPS, the proportion of hippocampal and striatal astrocytes expressing genes associated with the inflammatory phenotype and CXCL10 mRNA increased, suggesting that age and inflammation exert a cumulative effect on reactive astrocyte populations [21]. In age-associated neurological diseases, like PD and AD, dopaminergic neurons can display increased pro-inflammatory NLRP3 inflammasome activity [127]. In experiments utilizing activating mutations, mice with increased NLRP3 expression displayed advanced progression of motor deficits and increased levels of A1-like markers in striatal tissue [127].

## Reactive astrocyte responses to injury

### Spinal cord injury

Spinal cord injury (SCI) is a devastating and irreversible injury that affects roughly 300,000 people worldwide each year. SCI results in a dysregulated microenvironment within the lesion site. This is largely driven by the immediate response of microglia and resident astrocytes that release diverse signaling molecules to provide auto-regulatory feedback or establish molecular microglia-astrocyte conversion [59]. SCI progression includes both a primary and secondary injury. Primary injury refers to a mechanical injury to the spinal cord caused by a vertebral fracture or dislocation, while the secondary injury occurs within three different stages: acute, subacute, and chronic, resulting in different pathological characteristics [73]. In an acute injury, astrocyte activation, vascular damage, ion imbalance, neurotransmitter accumulation, free radical formation, and inflammation occur quickly after SCI [73]. In the subacute phase, apoptosis occurs as well as demyelination of surviving axons and matrix remodeling [73]. Lastly, in the chronic stage, scar formation and failure of axonal connections transpires [73]. Recent pathological studies have focused on the role of astrocytes in glial scar formation post-SCI. Glial scars serve seemingly paradoxical roles, in that they isolate damaged regions but also form a mechanical barrier to nerve fiber and primordial cell regeneration [73]. Glial scar formation is a highly dynamic process facilitated by multiple cell types, including astrocytes, microglia, and oligodendrocyte precursor cells [59, 73]. Spinal cord injury results in astrocyte reactivity, inevitably impacting functional recovery.

#### Neuroinflammatory astrocytes post-SCI

The complete role of astrocytes in SCI is not yet clear; however, they are known to play critical roles in glial scar formation. Indeed, the absence of astrocytes in the context of SCI results in a reduction in axon regrowth despite stimulation, suggesting that glial scar-forming astrocytes facilitate recovery of damaged neural circuits [5, 109]. Like in other neurodegenerative diseases, SCI is accompanied by persistent inflammation; thus, the pathological contributions of the pro-inflammatory phenotype to SCIs are worth considering [103].

#### JAK/STAT pathway

While the exact mechanisms underlying astrocyte polarization responses have yet to be fully elucidated, many pathways A1-like astrocytes may play a role in have been identified. STAT3 is a transcription factor within the Jak-STAT signaling family and has been shown to transduce signals for several cytokines and growth factors implicated in the injury response [55]. The activation of STAT3 by phosphorylation increases markedly in astrocytes, microglia, endothelial cells, and neurons shortly after CNS injury, and is particularly important in reactive astrocytes [55, 66, 103]. STAT3 phosphorylation and activation by Janus kinases (JAKs) have been demonstrated in a variety of neurodegenerative disease models and has been shown to play a role in damage repair, cell survival, and scar formation [73, 103]. Deletion of STAT3 can switch the neuroinflammatory A1 phenotype to the neuroprotective A2-like phenotype. JAK/STAT3, together with MAPK and NF-κB pathways, are significant initiators and modulators of astrocyte reactivity [73]. Furthermore, it is suggested that reactive astrocytes cause lesion repair and inflammation recovery through the STAT3 pathway [73].

#### Notch signaling pathway

The Notch signaling pathway is an evolutionarily-conserved mechanism that regulates cellular differentiation, proliferation, and apoptosis, which involves the binding of membrane Notch receptors to ligands that are expressed on adjacent cell membranes and subsequent downstream signaling events that activate transcription factors, effector molecules, and regulatory molecules [103]. As this highly-conserved pathway regulates proliferation of reactive astrocytes after traumatic brain injury and stroke, Qian et al. [103] further investigated the role of Notch signaling on the activation of pro-inflammatory astrocytes after spinal cord injury [103]. Numerous studies have demonstrated an association between Notch and Stat3 signaling. Importantly, changes in Notch expression can alter STAT3 phosphorylation and activity, which suggests Notch signaling can alter astrocyte phenotype by affecting STAT3 activity and function [103]. There has been evidence that A1-like reactive astrocyte numbers are increased at the lesion site following SCI in rats, and the phenotypic transition is dependent on the Notch-Stat3 axis. Reactive astrocytes can induce neuronal apoptosis and axonal damage through Notch-dependent release of pro-inflammatory factors [103].

#### NF-κB pathway

Nuclear transcription factor-κB (NF-κB) can be activated by stimuli associated with damage and in turn can promote expression of pro-inflammatory cytokines and is believed to play a central role in most inflammatory responses. After damage to the CNS, the resident cell types that activate NF-κB responses have not been completely characterized but are likely to include astrocytes [55]. Recently, activation of the NF-κB pathway has been implicated in the pathological changes in the CNS and that inhibition of this pathway helps limit disease progression [73]. The NF-κB signaling pathway is also associated with astrocyte polarization [135]. Though the exact mechanism is unknown, it is suggested that reactive astrocytes cause pathological damage through this pathway [73].

#### TGF-β signaling

As stated previously, reactive astrocytes build a glial scar to contain damage and protect surrounding tissue, but this is also likely to impede the regeneration of axons [55, 109]. After vascular damage with disruption of the BBB, the soluble blood coagulation protein fibrinogen leaks into the CNS and is converted to insoluble fibrin by thrombin [55]. Furthermore, the effect of fibrinogen on astrocytes is likely to be indirect through the TGF-β pathway and can act as carrier of latent TGF-β to sites of injury [55]. TGF-β signaling is an important factor of reactive astrogliosis following SCI and has been found to be a key upstream trigger of chondroitin sulfate proteoglycan (CSPG) expression in glial scar formation [73].

### Chronic pain

Chronic pain, defined as pain that lasts longer than 3 months, includes symptoms such as unpleasant sensory and emotional experiences, possibly coupled with actual tissue damage [72]. Around one third of Americans are affected by chronic pain and unfortunately cases are rapidly rising each year [72]. Pain has historically been viewed from the “neural center” standpoint, in which spinal neuronal pathways regulate “normal” pain signals that become overactive during chronic pain [72]. However, it has recently been proposed that spinal glial cells, specifically astrocytes, are also involved in the regulation of pain [72].

Despite their roles in homeostasis, following noxious stimulation and nerve injury, the phenotype, functions, and gene expression of astrocytes can elicit a significant change in the form of reactive astrogliosis [72]. The conversion of astrocytes from normal to reactive phenotypes encompasses a variety of intercellular and intracellular signaling pathways [72]. Signaling molecules that activate naïve astrocytes can be released by several cell types, including neurons and glial cells such as microglia, oligodendrocytes, and astrocytes, as well as other inflammatory cells [72]. There are many signaling molecules involved in or associated with the phenotypic transformation of astrocytes, including pro-inflammatory cytokines (IL-1β, TNF-α, and IL-6), gene transcription factors (STAT3), extracellular signal-regulated kinase 1/2 (ERK1/2), oligodendrocyte transcription factor 2 (OLIG2), Smaand Mad-related protein (SMAD), and G1 to S phase transition 1 (GSPT1), and various other proteins (GFAP, connexins, and AQP4) [63] (Fig. 2). While chronic pain is an increasingly relevant issue, the role astrocytes may play has yet to be significantly characterized.

#### Gp130-JAK-STAT3 signaling pathway

Gp130 cytokines are involved in the regulation of numerous biological processes, including hematopoiesis, immune response, inflammation, cardiovascular action, and neuronal survival [133]. These cytokines share glycoprotein 130 as a common signal transducer in their receptor complex and typically activate STAT3 [133]. Activated STAT3 translocates to the nucleus and has been shown to influence the transcription of GFAP, AQP4, connexins, and inflammation-related genes like nitric oxide synthase 2 (NOS2) [72]_._ Inhibition of astrocytic STAT3 reduces proliferation and migration following spinal cord injury; therefore, it is likely that GP130-JAK-STAT3 signaling mediates astrocytic proliferation, hypertrophy, migration, and glial scar formation [72]. In a study modeling neuropathic pain, it was indicated that the astrocytic JAK-STAT3 signaling pathways was found to be critical for astrocyte proliferation and maintenance of neuropathic pain [124]. Moreover, inhibition of this pathway has been shown to relieve pain from a simple touch, known as tactile allodynia, induced by spinal nerve injury, indicating the contribution of the JAK-STAT3 pathway in the progression of significant neuropathic pain by regulating astrocyte activation [72].

#### Notch-OLIG2 signaling pathway

Notch is an upstream signaling molecule of OLIG2 that promotes the translocation of OLIG2 to the nucleus of reactive astrocytes [82]. γ-Secretase inhibitor (GSI) has been found to not only inhibit Notch signaling, but also decrease nuclear translocation of OLIG2, resulting in the reduction of reactive astrocyte proliferation [110]. In a study performed using a model of sciatic nerve chronic constriction injury-induced neuropathic pain, it was suggested that the inhibition of astrocyte activation markers like GFAP and OLIG2 relieved chronic constriction injury-induced mechanical hyperalgesia, suggesting that NOTCH-OLIG2 signaling may play an important role in astrocytic proliferation and neuropathic pain and should be investigated further [10].

#### TGFβ-RGMa-SMAD signaling pathway

TGFβ is an important regulator that induces reactive astrogliosis and glial scar formation and is rapidly upregulated after CNS injury. Additionally, TGFβ is known to activate the SMAD family of transcription factors in astrocytes [63]. Through the TGFβ-SMAD3 signaling pathway, vimentin, actin, and GFAP expression in astrocytes increases, significantly contributing to scar formation after injury as well as delays in nerve recovery [130]. Repulsive guidance molecule a (RGMa) is a newly discovered membrane protein that mediates reactive astrogliosis and glial scar formation by regulating the TGFβ1-SMAD2/3 pathway [72]. In a study about neuropathic pain following spinal cord injury, it was found that RGMa is upregulated after rat and human spinal cord injury. Furthermore, treatment with an RGMa antibody attenuated the associated neuropathic pain [88]. This suggests possible therapeutic potential of targeting TGFβ-RGMa-SMAD signaling pathway, and indeed other astrocyte activation pathways, to decrease neuropathic pain.

## Reactive astrocytes in neurodegenerative and neurological diseases

### Neurodegeneration

Given the critical roles astrocytes play in ensuring proper CNS function, including maintaining BBB integrity, phenotypic alterations can significantly contribute to neurological impairments. A1-like astrocyte populations have been shown to arise with normal aging and are further increased in neuroinflammatory contexts; accordingly, reactive astrocyte abundance is associated with several infectious and neurodegenerative disorders [21, 75]. The first research into the newly-characterized A1-like astrocytes explored their role in the more common neurodegenerative disorders, including Alzheimer’s disease (AD), Parkinson’s disease (PD), Huntington’s disease (HD), amyotrophic lateral sclerosis (ALS), and multiple sclerosis (MS) [21, 75, 76]. Indeed, the initial work by Shane Liddelow and Ben Barres that described A1/A2 polarization in 2017 confirmed the translational relevance of their findings using human post-mortem brain tissue samples from relevant regions of neurodegeneration in these disorders [76]. In situ hybridization and co-immunofluorescent staining methods to analyze AD, PD, HD, ALS, and MS samples revealed a high degree of co-localization of the astrocyte markers S100β and GFAP and the A1 marker C3 in disease-relevant brain regions, indicating that the astrocytes present had adopted a pro-inflammatory phenotype [76]. In healthy tissue, the proportion of astrocytes that could be classified as A1-like astrocytes is low, typically ranging from 10 to 20% of the local astrocyte population, depending on the brain region [76]. A1-like astrocytes are not so rare in neurodegenerative disorders; indeed, up to 60% of astrocytes in regions of the brain affected by neurodegeneration in these chronic disorders are of an A1-like phenotype, effectively taking over the astrocyte population [76]. When they examined the presence of reactive astrocytes within demyelinating lesions across different MS stages, they found that A1-like polarization correlated with disease progression [76]. Neurotoxic A1-like astrocytes have altered, cytotoxic functions, secrete pro-inflammatory signals, and promote neuroinflammation, contributing to neurodegeneration [21, 75, 76].

The primary goal of neuroinflammation is to defend against pathogenic infiltration into the CNS; however, neuroinflammation is also a hallmark of neurodegenerative diseases. Typically, there is a low-level, chronic expression of pro-inflammatory signals throughout the course of neurodegeneration, and this long-term inflammatory state may contribute to disease progression [66]. Neurodegenerative diseases like AD and PD are characterized by toxic aggregates of pathological proteins, such as tau and amyloid-β in AD and α-synuclein in PD. Astrocytes and microglia can function cooperatively to process toxic protein deposits [105]. Specifically, co-cultured human iPSC-derived astrocytes and microglia exposed to amyloid-β and α-synuclein engulf the protein aggregates, then transfer the internalized protein between each other through tunneling nanotubes [105]. The use of human iPSC-derived astrocytes is significant, as rodent-derived iPSCs do not necessarily share the same gene expression profiles [9]. This cell-to-cell transfer of pathological protein is consistent with the organized manner these cells clear debris [105]. Larger aggregations tend to be degraded by microglia, while astrocytes, which can act as antigen-presenting cells, are more likely to process the smaller or monomeric proteins [105]. However, this mechanism is insufficient to prevent the progression of diseases like AD and PD, and the clearing capacity of these cells becomes overwhelmed. Aggregations of pathological proteins further damage nearby cells, prompting the release of pro-inflammatory cytokines, which serve as signals that induce microglia to adopt an inflammatory phenotype [66]. These activated microglia go on to mount an inflammatory response, which includes astrocyte polarization via secretion of TNF-α, IL-1α, and C1q [76]. Increased levels of inflammatory markers, like TNF-α or IL-1β, have been detected in the CSF of individuals with neurodegenerative disorders, and these levels correlate to degree of cognitive impairment, emphasizing the importance of neuroinflammation in neurodegenerative disease progression [16, 66].

### Alzheimer’s disease (AD)

AD is a progressive neurodegenerative disorder that currently effects just under 50 million people. With advances in medicine prolonging the average human lifespan, the incidence of AD is expected to rise to 115 million in the next 30 years [36, 58]. As the leading cause of dementia, AD is one of the most commonly-studied neurodegenerative disorders. The primary pathological features found in AD include extracellular deposits of abnormal proteins termed amyloid-β (Aβ) that form destructive amyloid plaques in the brain tissue, and intraneuronal aggregates of misfolded and hyperphosphorylated Tau protein that form toxic neurofibrillary tangles. The accumulation of these pathogenic proteins over time is associated with synapse loss, neuronal death, and chronic neuroinflammation [8, 36, 58]. The initial pathology appears to be localized to the entorhinal cortex and hippocampus, structures associated with memory formation [8, 13]. Difficulties with memory and executive functioning expand to include other cognitive dysfunctions, including aphasia. People living with moderate AD often lose the ability to complete tasks with multiple steps, learn new information, recognize faces, and consolidate long-term memories [7]. Neuropsychological symptoms also arise, frequently resulting in disorientation, hallucinations, and paranoia [7]. Increasing damage and substantial cortical atrophy results in the eventual loss of communication abilities, as well as the loss of physical functions like walking and even swallowing. As a progressive neurogenerative disorder, the complications of AD ultimately prove fatal.

Prior to the introduction of the A1/A2 classification paradigm, non-proliferative reactivation had been observed in astrocytes in AD. Analysis of temporal cortex samples from symptomatic AD patients revealed increased levels of reactivity markers, specifically major histocompatibility complex II (MHCII) for microglia and GFAP for astrocytes [108]. However, the levels of constitutively-expressed markers, ionized calcium-binding adaptor molecule 1 (IBA1) in microglia and aldehyde dehydrogenase 1 L1 (ALDH1L1) in astrocytes, did not increase indicating the reactivation response was phenotypic in nature rather than solely proliferative [108]. Furthermore, cortical NF-κB and C3 expression levels are significantly higher in the context of AD, both in human patients and transgenic mouse models [74]. Both in vitro and in vivo, C3 upregulation is correlated with Aβ accumulation, a hallmark of AD. Further investigation indicates that Aβ exposure activates the NF-κB pathway in astrocytes, with one of the transcriptional targets being C3 [74] (Fig. 1e). Astrocytes release the upregulated C3, which binds to the C3a receptor (C3aR) on neurons. Increased C3-C3aR signaling dysregulated intraneuronal calcium homeostasis, ultimately disrupting dendrite morphology, reducing synaptic density, and impairing excitatory synaptic transmission in the context of AD [74] (Fig. 2). These findings are consistent with a pathological role for some reactive astrocytes, which display NF-κB pathway activation and increased C3 expression [76, 135].

AD has quickly become a commonly used model to study A1-like astrocytes. Since their characterization, a variety of mechanisms by which A1-like astrocytes contribute to AD pathology have been described. The link between A1-like astrocytes and AD appears to begin at the genetic level. Among the 40 or so loci that may possess alleles associated with an increased risk of developing AD is the *SPI1* gene which encodes PU.1, a transcription factor that regulates myeloid cell development and differentiation [101]. For example, in response to injury or insult in the brain, PU.1 expression may be upregulated, allowing it to facilitate transcriptional changes in microglia and resident macrophages [101]. In AD there are abnormally high levels of PU.1 expression, and several of the genes it controls are also associated with AD development [101]. One consequence of this altered microglia response is altered astrocyte activation. When microglia with AD-like PU.1 overexpression are exposed to LPS, they are more efficient at inducing polarization in astrocytes, as evidenced by increased A1-specific transcriptional markers when exposed to LPS-stimulated MCM from PU.1 overexpressing microglia, compared to wildtype microglia [101].

Interestingly, it appears that microglia are not the only mechanism by which A1-like astrocytes are activated in the context of AD. One model of AD uses trimethyltin (TMT) intoxication, in which the resulting hippocampal degradation gives rise to molecular and behavioral signatures of AD in rodents [28]. When applied to primary cortical astrocytes in vitro, TMT disrupts intracellular Ca^2+^ homeostasis by causing an influx of Ca^2+^ through L-type voltage-gated ion channels [28]. Subsequently, the mitochondrial membrane becomes depolarized, levels of ROS and NOS increase, and A1-associated pro-inflammatory pathways are activated (Fig. 1f). The C3 + A1 phenotypic switch that occurred in this model of AD was also accompanied by morphological changes including retraction of cellular processes [28]. Other in vitro and in vivo models have also been useful in cataloging the roles of A1-like astrocytes in AD. Aβ has been shown to act in vitro similarly to a pro-inflammatory cytokine and even at low concentrations can be substituted for C1q in the TNF-α and IL-1α cocktail that induces polarization to a pro-inflammatory phenotype [70] (Fig. 1e). Further research has demonstrated that Aβ-treated microglia-conditioned media not only promoted A1-like polarization, but also upregulate astrocytic glycolysis [145] (Fig. 2). Interestingly, changes in glucose metabolism are characteristic of early AD, and the role of glycolysis in astrocyte activation in both AD and other pathologies requires continued investigation [145]. Overall, these experiments suggest that at least one of the pathological features underlying AD, Aβ aggregation, can itself activate astrocytes and promote a phenotypic polarization, potentially explaining the high proportion of A1-like astrocytes in post-mortem human brain tissue of individuals with AD [76] (Fig. 1e). As AD most often appears in elderly populations, this issue is likely compounded by the natural accumulation of A1-like astrocytes as the brain ages [21].

The neurotoxic and pro-inflammatory phenotype of A1-like astrocytes likely contributes to the progression of AD pathology. Astrocyte-derived exosomes (ADEs) obtained from the blood plasma of individuals living with AD were found to contain high levels of a variety of complement proteins, including C3, and decreased levels of complement regulatory proteins, compared to ADEs from individuals without AD [34] (Fig. 2). This suggests the astrocytes adopted an A1-like phenotype. When analysis was applied to a longitudinal investigation, samples from patients in early pre-clinical stages had complement levels comparable to the control group, and as the disease progressed and symptoms developed, complement levels in ADEs increased [34]. A1-like astrocytes may also exert pathological effects in the context of AD by contributing to signaling dysfunction [8]. In one study that looked at hippocampal and entorhinal cortical samples from a rat model of AD and non-AD control, C3 + reactive astrocytes significantly upregulated expression of serine racemase (SR) [8]. SR catalyzes the synthesis of D-serine, a co-agonist for the neuronal NMDA receptor (NMDAR), and its overexpression by A1-like astrocytes was associated with increased NMDAR activation and associated excitotoxicity [8]. Consistent results were observed in human post-mortem brain tissue samples obtained from previously diagnosed AD patients, indicating reactive astrocyte-mediated excitotoxicity may be another mechanism by which neurotoxic astrocytes contribute to neurodegenerative disease [8].

A major challenge to treating AD is the lack of any easily-detectable biomarkers in living patients. AD is diagnosed via a battery of neuropsychological assessments and can only be definitively confirmed at autopsy where Aβ and pTau aggregates can be identified in brain tissue [36]. Neuroimaging techniques, including PET and MRI, in combination with CSF analysis can provide a more thorough examination, but there are major financial and access barriers to these invasive methods [36]. Symptoms do not appear until amyloid plaques and neurofibrillary tangles have elicited enough irreversible tissue damage, rendering early treatment impossible. The recent discovery of reactive astrocytic phenotypes may ultimately provide a method of pre-symptomatic identification of AD, potentially by analyzing an unlikely location: the retina. Aβ and pTau aggregates were detected via immunostaining of post-mortem retinal slices from patients with AD, as well as associated increased levels of IL-1β, which co-localized with microglial Iba1, and C3 colocalized with astrocytic GFAP [36]. This could pave the way for future diagnostic applications of non-invasive retinal scans.

Supporting the hypothesis that different reactive astrocyte polarization dynamics may in some way complement each other, one study identified the presence of both phenotypes in the context of AD [58]. Astrocyte populations were assessed using a cohort of post-mortem human samples from diagnosed and neuropathologically-confirmed cases of AD at similar progression stages and non-AD controls [58]. Immunostaining with C3 was used to indicate A1-like neuroinflammatory astrocytes, while S100A10 was used to indicate neuroprotective A2-like astrocytes [58]. The number of A1-like cells was higher than A2-like cells in both control and AD, suggesting that the two phenotypes are not evenly balanced [58]. In AD, the density of A1-like cells was significantly increased in certain tissues, specifically in the upper cerebral cortex. Interestingly, the density of A2-like cells was also significantly increased in AD, in the upper and lower cortex as well as the white matter [58]. The researchers suggest that a neuroinflammatory response occurs in AD, in which quiescent astrocytes become A1-polarized and pre-existing age-associated A1-like astrocytes become more active [58]. Reactivity associated with pathogenic protein accumulation may explain why reactive astrocyte growth is more localized rather than widespread. Subsequent neuroinflammation and damage could elicit a generalized A2-like polarization signal across a larger tissue area to provoke a neuroprotective response [58]. More research will be needed to investigate this potential mechanism and tease out the interplay among neuroinflammatory A1-like and neuroprotective A2-like astrocytes.

### Parkinson’s disease (PD)

Parkinson’s disease (PD) is the second most common neurodegenerative disorder and affects 2% of all people over the age of 65 [136]. The risk increases if an immediate family member also has PD, suggesting a hereditary link in some cases, while several spontaneous genetic mutations are suspected of being causative in other cases [23, 125]. PD is classified as a degenerative type of parkinsonism, which describes any disorder that causes slowed movement or bradykinesia, rigidity, limb dystonia, tremors, and postural instability [23]. Non-motor neuropsychological symptoms like emotional dysregulation, sleep disturbances, dementia, and psychosis, are often present prior to diagnosis and become more severe as the degeneration progresses [23, 125]. While not directly fatal, complications that arise secondary to the pathological changes in PD can be life-threatening.

The symptoms of PD arise due to loss of dopaminergic neurons in the basal ganglia, particularly in the substantia nigra, and a corresponding dopamine signaling deficiency [125, 136]. A key pathological feature of PD is the presence of cytotoxic Lewy bodies, intracellular inclusions containing accumulated misfolded α‐synuclein [136]. Neuroinflammation is another hallmark of PD. Post-mortem samples display significant levels of inflammatory microglia activation [136]. In addition to secreting pro-inflammatory factors like NO and TNF-α, activated microglia in PD may also promote T cell-facilitated neuronal cell death [4, 14, 47, 85, 136]. The dopaminergic neurons themselves display upregulation of the NF-κB pro-inflammatory pathway in the context of PD [45, 75]. Using a mouse model of PD, AQP4 deletion was found to significantly exacerbate neuronal cell death and disease progression [136]. This was also associated with increased microglial activation, neuroinflammation, and downregulation of astrocyte-secreted TGF-β [136.

PD was one of the first neurodegenerative disorders that inflammatory A1-like astrocytes were initially identified in, with astrocytes staining positive for C3 in post-mortem substantia nigra tissue samples collected from people who had been diagnosed with PD [76]. The proportion of astrocytes that could be characterized as A1-like astrocytes, positive for both GFAP and C3, was nearly 20-fold higher in human PD samples compared to non-diseased controls [76]. The value and applicability of identifying A1-like astrocytes and their pathological contributions in a given disease is demonstrated in much of the current PD research. It had been previously observed that glucagon-like peptide-1 receptor (GLP-1R) agonists had neuroprotective effects in neurodegenerative diseases like PD; however, the mechanism was unknown [140]. The BBB-permeant GLP-1R agonist NLY01 was found to inhibit microglia from secreting the inflammatory mediators that elicit pro-inflammatory polarization, TNF-α, IL-1α, and C1q [140]. When tested in a murine model of PD, repression of microglia-mediated astrocyte reactivity prevented dopaminergic neuron loss and resulting behavioral dysfunction [140]. This further supports a role for astrocyte polarization in the progression of PD and offers a potentially effective avenue of symptom management to explore.

### Huntington’s disease (HD)

Huntington’s Disease (HD) is a herditary neurodegenerative disorder that arises due to elongated CAG repeats in the gene that encodes the Huntingtin protein [104]. The age of onset is linked to the length of these repeats, with most symptoms of involuntary choreatic movement typically first developing during middle age, or earlier in cases of juvenile HD [104]. As the disease progresses over the course of roughly 15–20 years, psychomotor impairment, neuropsychiatric disturbances, cognitive decline, and dementia develop. Pneumonia resulting from complications of HD is the most common direct cause of death [99]. The huntingin protein appears to be supportive of synaptic function. While the exact etiology is still being investigated, the mutated huntingtin protein becomes misfolded and forms inclusion bodies that may be neurotoxic [104].

Increased populations of A1-like reactive astrocytes, as indicated by C3 and S100β double-staining, have been identified in post-mortem tissue samples of the caudate nucleus obtained from people diagnosed with HD [76]. Such samples are most likely representative of more advanced cases of HD. Gene expression analysis of astrocyte markers at different stages of disease progression detected upregulation of pro-inflammatory astrocyte markers in stage 3, but not in earlier stages [25]. Therefore, the significant neuronal death that occurs during stages 1 and 2 is not likely a consequence of astrocyte polarization [25]. Inflammatory astrocytes do not appear to play as significant a role in the development of HD, but may be relevant for the later stages of pathology. How the presence of A1-like astrocytes may or may not contribute to advanced HD has not yet been elucidated.

### Amyotrophic lateral sclerosis (ALS)

Amyotrophic lateral sclerosis (ALS) is a progressive neurodegenerative disorder characterized by upper and lower motor neuron (MN) loss in both the cortex and spinal cord [143]. Symptoms typically include muscle weakness that progresses to paralysis, dysphagia, and ultimately death within a few years, typically due to respiratory complications [143]. ALS pathogenesis appears to involve glutamate excitotoxicity, abnormal mitochondrial function, increased levels of ROS, and axonal defects [143]. ALS is most often sporadic, with only 5–10% of cases being familial. Like AD and PD, ALS is associated with toxic protein aggregates; however, the protein in question can vary. Some cases have been linked to mutations of the superoxide dismutase 1 (SOD1) gene, which results in misfolded enzyme aggregation in MNs, while other cases involve aggregation of the TDP-43 protein [100, 143]. Ultimately, the underlying cause of ALS in most cases remains unknown.

While the exact mechanisms remain unclear, MN death in ALS is not cell-autonomous [48]. As such, the role of astrocytes in disease pathology has recently gained attention. Neurotoxicity is not the only detrimental effect of protein aggregation in the context of neurodegenerative disease. Reactive responses may also be induced by the loss of function that accompanies the sequestration of proteins important for normal cellular function. TDP-43 inclusions within neurons and glial cells are found in over 90% of sporadic ALS cases [100]. These intracellular aggregates specifically form in the cytoplasm and are associated with a corresponding loss of intranuclear TDP-43. TDP-43 is a nucleic acid-binding protein that serves a large variety of regulatory roles in CNS cells [100]. Using a mouse model with an astrocyte-specific TDP-43 deletion mutation to represent an abnormal loss of function, astrocytes deficient for this protein adopted a transcriptional profile resembling that of pro-inflammatory astrocytes (Fig. 1g). Additionally, the presence of these A1-like astrocytes resulted in upregulation of C1q expression by microglial cells [100]. This result suggests that the loss of functional TDP-43 in ALS may induce an A1-like reactive astrocyte response, indicating that this pro-inflammatory response may serve a regulatory role [100]. Interestingly, while the presence of these A1-like TDP-43-depleted astrocytes was associated with the development of motor deficits, the astrocytes themselves were not directly neurotoxic to motor neurons. Instead, the presence of A1-like cells resulted in a loss of mature oligodendrocytes, suggesting a multi-cellular mechanism for TDP-43-mediated neurotoxicity in ALS [100]. Confirming the relevance of these findings, post-mortem tissue harvested from the motor cortex of humans diagnosed with ALS was found to have significantly increased populations of reactive astrocytes, as indicated by C3 and S100β double-staining [76].

There is evidence to suggest that ALS pathology also includes dysregulation of AQP4 expression resulting in impaired BBB integrity and function [148]. In a SOD1-overexpressing transgenic rat model of ALS, increased AQP4 mRNA and protein expression was detected in spinal cord grey matter [92]. There is evidence for astrocytic endfeet swelling and impaired BBB integrity due to both increased and decreased AQP4 expression, possibly suggesting that a delicate balance must be maintained for proper function [148]. Atrophic muscle tissue from people with ALS was found to have decreased AQP4 expression, suggesting this dysregulation may be secondary to muscle denervation [50]. Most of the literature has focused on increased AQP4 expression in the context of ALS; thus, the decreased AQP4 expression observed in A1-like pro-inflammatory astrocytes may have arisen as a compensatory response. Ultimately, significantly more research is needed to further elucidate the role of astrocytic AQP4 dysregulation in ALS and what role astrocyte polarization may play.

### Multiple sclerosis

Multiple Sclerosis (MS) is an autoimmune disease in which the protective myelin that ensheath neuronal axons is progressively degraded. This chronic demyelination, primarily facilitated by T helper cells, results in axonal damage, impaired neuronal signaling, and neuronal death [61, 83]. Myelin, produced by oligodendrocytes in the CNS, insulates regions of the axon from the extracellular ion gradient and establishes saltatory conduction, in which the action potential flows down the axon uninterrupted in regions shielded by myelin until it reaches the interspersed Nodes of Ranvier. The membrane in these exposed regions between the myelin sheaths is depolarized, and the signal continues to propagate in this way. As such, myelin allows an action potential to efficiently travel uninterrupted along the axon [33]. MS can have varied disease presentation, but over 80% of cases are considered to be Relapsing Remitting MS (RRMS), in which neurological symptoms alternate with periods of remission, the duration of which can be days or years [61]. The most common symptoms of MS include dysfunction of sensation and perception systems like vision, as well as motor skills and balance, resulting in severe disability. New symptoms can arise with each relapse, and the trigger for these relapses are not well understood but appear to be associated with physiological stressors, such as infection or pregnancy [61]. Likewise, the mechanism underlying the temporary cessation of disease progression is not clear, but the alleviation of some symptoms during these remission periods may be due to re-myelination, ultimately resulting in a cycle of accumulating damage and attempted repair [61]. When left untreated, or in advanced cases, RRMS can progress to Secondary Progressive MS. In the progressive stage of the disease, the disability states are more stable and can result in complications with muscle control that can prove fatal [61].

MS progression can also be described in terms of inflammatory staging of lesions based on histological criteria [76]. Using this, the original report describing the A1 astrocyte phenotype demonstrated that the proportion of astrocytes characterized as A1-like astrocytes increased proportionally with the severity of the lesions [76]. Supporting this finding, when cerebrospinal fluid was collected from people living with MS of varying progression, correlating biomarkers of microglia activation were identified, including DSG2 and TNFSF25 [83]. Furthermore, activated astrocyte markers were also present at levels correlated with severity; specifically, markers that are also representative of inflammatory reactive astrocytes such as SERPINA3 and CLIC1 [83 (Fig. 2). This is consistent with the understanding of MS as a disease of persistent chronic inflammation, likely sustained by glial activation. As an example of this, excessive glial scar formation has also been suggested as contributing to neuronal dysfunction in MS. One recent investigation explored this by looking at expression of proteins associated with glial scar formation [53]. Within graded MS lesions, the associated astrocytes displayed significantly increased levels of the Ca^2+^-permeable nonselective cation channel TRPM7. This finding was replicated in vitro with astrocytes treated with pro-inflammatory stimuli [53]. Cultured inflammatory A1-like astrocytes with increased TRPM7 expression were also found to have elevated levels of CSPG glial scar components [53]. In a co-culture of neurons and TRPM7-overexpressing astrocytes derived from rodent cortex, the increased production of CSPGs appeared to restrict neuronal outgrowth, which could ultimately contribute to neuronal damage in demyelinating disease [53].

Research into MS pathology is frequently performed using a rodent model of experimental autoimmune encephalomyelitis (EAE). Using this model to understand the role of neurotoxic astrocytes in MS has yielded an array of novel findings. A microfluidic RT-qPCR screen of both astrocytes and microglia in a mouse EAE model identified upregulation of pro-inflammatory genes, in both the relapsing and remission stages, as well as evidence of dysregulated endocannabinoid signaling in microglia [87]. The neuroinflammation present in MS and EAE can also be linked to activation of the microglial NLRP3 inflammasome, an innate immunological signaling response comprised of apoptotic NLRP3 proteins that process IL-1β and IL-18 [44]. qPCR analysis of rodent astrocyte cultures exposed to IL-18 detected significant upregulated of inflammation–associated genes and down-regulated repair–associated genes, and western immunoblotting and immunofluorescence confirmed increased C3d expression in treated astrocytes, suggesting that IL-18 may induce astrocyte polarization [44] (Fig. 1h). Neurons cultured with the IL-18-induced reactive astrocyte-conditioned media, displayed a loss of synaptic density. As pre-treatment with a C3R inhibitor reduced this effect, the synapse loss was attributed to astrocytic C3 [44]. Using an EAE rodent model, investigators found that hippocampal synapse loss could be ameliorated with an NLRP3 inflammasome inhibitor, corresponding with reduced development of memory and cognitive deficits in advanced stages of disease. Finally, it was confirmed that NLRP3 inflammasome inhibitor treatment also prevented the rise in C3 representing A1-like polarization [44]. Overall, this suggests that in MS the microglial NLRP3 inflammasome becomes activated and induces polarization, and the subsequent rise in astrocyte C3 contributes to disease pathology. More recent work utilizing RNA-seq technology to profile the cellular environment surrounding MS lesions revealed inflammatory astrocytes and increased expression of signals that induce astrocyte polarization, such as C1q [1].

The relationship between astrocytes and oligodendrocytes in MS have also been investigated using EAE rodent models. In non-pathological contexts, astrocytes form gap junction channels with the myelin-producing oligodendrocytes, using connexin 43 (Cx43) and connexin 47 (Cx47), respectively [146]. Notably, expression of oligodendrocyte Cx47 within MS plaques progressively decreases. To understand the consequences of this phenomenon, an inducible conditional Cx47 knockout mouse model was developed [146]. The complete absence of Cx47 expression was associated with more extensive demyelination and advanced overall EAE disease progression. This suggests that the loss of Cx47 that occurs over the course of the disease contributes to further disease progression and exacerbation [146]. Additionally, microarray analysis indicated that the loss of Cx47 expression was correlated with pro-inflammatory microglia activation. This may underlie the immunohistochemical finding that the neurotoxic astrocyte marker C3 was also significantly increased, particularly in the acute stages of the disease [146]. This suggests that the loss of oligodendrocytes and Cx47 as demyelination progresses triggers a neuroinflammatory response in which astrocytes are polarized to a pro-inflammatory phenotype.

### Other neuropathologies

#### Familial Danish Dementia (FDD)

In neurodegenerative diseases like AD, a common complication is cerebral amyloid angiopathy (CAA), in which damage is incurred due to cerebrovascular amyloid deposition [123]. Familial Danish Dementia (FDD) is another neurodegenerative disease in which CAA is a prominent etiological feature. While AD is characterized by parenchymal Aβ deposition, FDD is associated with leptomeningeal and cerebrovascular Danish amyloid (ADan) accumulation [123]. FDD appears to be the result of a major frameshift mutation in the BRI2 gene that ultimately produces an abnormal ADan with amyloid subunits, forming cerebrovascular-associated plaques and neurofibrillary tangles [123]. Adding to the growing understanding of glial cell reactivity involvement in parenchymal amyloid deposits, a recent study examined the relationship with vascular amyloid deposits in the early stages of the disease using an FDD mouse model (tg-FDD) [123]. In addition to dysregulated lipid processing and cholesterol accumulation, due to changes in gene expression of ApoE and TREM2, the study also described glial responses [123]. While vascular amyloids fail to significantly induce microglial reactivity or proliferation, it did induce astrogliosis. Interestingly, these reactive astrocytes were described as having an A1-like phenotype [123]. Furthermore, reactive astrocytes that displayed decreased TREM2 expression, appeared to mediate microglial homeostasis [123]. Further research into this lipid-ApoE-TREM2 pathway and the chronology of astrocyte-microglial interactions in CAAs can expand the body of knowledge related to A1-like astrocytes in neuropathology.

#### Bardet-Biedl syndrome (BBS)

A1-like reactive astrocytes have also been characterized in ciliary defects; specifically, Bardet-Biedl Syndrome (BBS). This autosomal recessive ciliopathy can arise from independent mutations in over 20 genes, many of which are associated with the BBSome protein complex, its assembly, and its localization. There is evidence to suggest that the BBSome complex contribute to ciliary protein trafficking and signaling [112]. In addition to obesity, cardiac and renal abnormalities, and retinal degeneration, BBS is also characterized by neurological symptoms, including hydrocephaly, region-specific volume loss, and intellectual disability. Given the observed association between defective ependymal ciliogenesis, neuroinflammation, and hydrocephalus, as well as the presence of reactive astrocytes in BBS mouse models, the contribution of reactive astrocytes to initial BBS neuropathology was investigated [112]. By comparing two BBS mouse models, a congenital knockout with hydrocephaly and an inducible knockout without hydrocephaly, the relationship between astrocyte reactivity and the development of hydrocephaly could be examined [112]. It was found that microglial activation was not required for astrocyte activation, and that A1- and A2-like reactive astrocytes were present, but differed in their molecular signatures, between the two models. Unlike the induced model, the congenital model displayed neuroinflammation and upregulation of several specific reactive astrocyte markers: GFAP; a Pan-reactive astrocyte marker, SERPINA3N; an A1 marker, and post-synaptic density 95 protein (PSD95); an A2-like marker (Table 1) [112]. These findings suggest that impaired BBSome function is associated with a range of dysregulated astrocyte reactivity that does not appear to be secondary to hydrocephalus [112]. Further investigations are required to elucidate underlying mechanisms and roles of reactive astrocytes in BSS and other potential ciliopathies.

#### Lysosomal storage disease (LSD)

Lysosomal storage diseases (LSD) encapsulate a wide range of rare, neurodegenerative disorders. Neuronal ceroid lipofuscinosis (NCL), a sub-type of LSD resulting from a variety of potential genetic mutations, are characterized by intraneuronal auto-fluorescent ceroid accumulation, as well as neuroinflammation [107]. A pediatric NCL subtype, infantile neuronal ceroid lipofuscinosis (INCL), is caused by a loss-of-function mutation in the *CLN1* gene. *CLN1* encodes palmitoyl-protein thioesterase-1 (PPT1), a lysosomal enzyme that facilitates depalmitoylation [107]. Protein functions are often regulated by post-translational modifications, one of which is S-palmitoylation, or the thioester linkage of a 16-carbon saturated fatty acid to a cysteine residue. For additional regulatory control, which has proven to be particularly relevant to the brain, dynamic S-palmitoylation may occur, in which palmitoylation is catalyzed by palmitoyl-acyl transferases (PATs or ZDHHCs) and subsequent depalmitoylation is catalyzed by lysosomal (PPT1) and cytosolic (acyl-protein thioesterase-1, APT1) thioesterases [107]. These modifications contribute to modulating hydrophobicity, which in turn influences membrane affinity and protein structure, as well as protein trafficking and protein–protein interactions. One important example of this process is the APT1 depalmitoylation of H-Ras at the cell membrane, the regulation of which contributes to cell proliferative functions [107]. To understand how neuroinflammation arises and contributes to the devastating disease progression in INCL, researchers used a mouse model with the same underlying mutation (Cln1-/-) [107]. In the brain, the loss of lysosomal PPT1 thioesterase was associated with decreased levels of specific PATs, the same PATs that catalyzed the activating palmitoylation of cytoplasmic APT1 thioesterase [107]. This resulted in a loss of membrane-localized APT1, and consequently, increased H-Ras. Unregulated by APT1, uninhibited H-Ras signaling resulted in microglial activation, proliferation, and pro-inflammatory signaling, including C1q expression. As expected, this provided the conditions suitable for the induction of reactive astrocyte polarization [107]. These findings may suggest that, in non-pathological contexts, astrocyte reactivity is kept at bay, in part, by the proper dynamic palmitoylation of H-Ras, and that in the context of INCL, the dysregulation of dynamic palmitoylation prohibits this regulation, resulting in both microglial and neurotoxic astrocyte activation. Further research is needed to investigate the conservation of this process across other NCLs or LSDs.

### Prion disease

Cellular prion proteins (PrP^C^) anchored to the cell surface are thought to be associated with cell signaling, particularly in neurons, synaptogenesis, cell adhesion, and to be protective against oxidative stress and apoptosis [131]. Encoded by the *PRNP* gene, these glycoproteins are comprised largely of α-helices when in their proper conformation. The unique feature of this constitutively expressed protein is that it can undergo a pathological post-translational conversion to a misfolded, β-sheet-rich form, termed PrP^Sc^, in reference to the founding discovery of the isoform in ovine scrapie disease [115, 128]. PrP^Sc^ interacting with soluble PrP^C^ induces the same conformational change via template conversion, facilitating transmissible misfolding. PrP^Sc^ may be secreted, transported across intercellular tunneling nanotubes, or released following cell death, and go on to seed other cells [35]. This infectious property is not restricted to host protein interaction, but also applies to contact with foreign human and compatible inter-species tissue containing PrP^Sc^. The PrP^Sc^ isoform is partially resistant to proteolytic degradation, fails to elicit a humoral response, and promotes chronic ER stress and unfolded protein response (UPR) activation [17, 115, 128]. Exponential self-propagation and PrP^Sc^ aggregation results in insoluble plaque formation, neuronal death, emergent tissue destruction, and given enough time, progressive atrophy of the brain so substantial the organ becomes cavity-ridden and sponge-like in appearance. These neurotoxic proteinaceous infectious particles, or prions, are the causative agent of several aggressively neurodegenerative and invariably fatal transmissible spongiform encephalopathies (TSE) [102].

Inherited mutations in the *PRNP* gene are responsible for some prion diseases, as in the case of Fatal Familial Insomnia (FFI) and Gerstmann-Sträussler-Scheinker disease (GSS). Prion diseases can also be acquired from inoculation or ingestion of prion-contaminated tissue, as is the cause of Kuru (human source) and variant Creutzfeldt-Jakob Disease (vCJD) (bovine source) [128]. Most cases, however, arise spontaneously, with sporadic CJD (sCJD) accounting for approximately 85% of all CJD prion diseases [126, 128]. PrP^Sc^ aggregation often progresses gradually, allowing the prion disease to go undetected for many years and significantly prolonging the pre-symptomatic period. The duration of time between initial prion seeding and symptom development is variable, with some cases of Kuru disease not arising until over fifty years after the initial exposure [22]. Unfortunately, the biological mechanism underlying this dormancy is not well understood.

Spongiform degeneration localization and symptom profiles vary between different prion diseases. The heritable prion disease FFI is associated with an autosomal dominant mutation in the *PRNP* gene; specifically, a missense mutation in codon 178 along with a methionine (M) present at polymorphic position 129 [128]. This rare prion disease has only been identified in several dozen families, with symptoms arising in adulthood. Prion-associated degradation in FFI is restricted to the thalamus, causing sleep/wake cycle disruption, lethargy, and significant weight loss [3]. Persistent insomnia that fails to effectively respond to treatment with sedatives results in symptoms associated with sympathetic nervous system activation and dysautonomia. Hallucinations, dementia, cognitive impairment, and psychomotor deterioration follow as the insomnia worsens in severity, with coma and death typically occurring after roughly 1 year [3]. CJD is the most prevalent of prion diseases and consists of several subtypes. Iatrogenic CJD results from the medical transplantation of infected tissue, while variant CJD resulted from ingestion of beef contaminated with bovine spongiform encephalopathic (BSE) tissue [128]. CJD can be inherited, with mutations in the *PRNP* gene, but most cases occur due to a sporadic PrP^C^ misfolding event (sCJD), occurring in approximately 1 in 1 million people [128]. In classical CJD, damage occurs throughout the cortex, resulting in a broad array of symptoms. While early signs commonly include pain and fatigue, progression of the disease gives rise to memory loss and muscle spasms [128]. sCJD can feature cortical blindness and progressive cerebellar ataxia. Akinetic mutism can develop in the most advanced cases, with death typically occurring less than six months following symptom onset [128].

Prion infection can arise and persist in neurons and astrocytes, both of which produce *PRNP* transcripts. In a mouse model of astrocyte-limited prion infection, neurodegeneration and disease progression is slowed, but still present, suggesting that astrocyte involvement may be a contributing factor in some cases [17]. The neuroinflammation observed in early stages of prion infection, characterized by increased levels of an array of pro-inflammatory cytokines, including IL1α, TNF-α, and CXCL10, is largely attributed to microglial activity [17]. Microglia activation occurs in response to aggregation of misfolded proteins during the pre-symptomatic phase of disease, which in turn promotes astrocyte reactivity; however, the specific reactive phenotypes that arise in the context of prion accumulation have only recently been the subject of investigation [17]. In experiments utilizing different mouse strains of scrapie, qRT-PCR was performed on CNS tissue collected at different timepoints [17]. As expected, neuroinflammation-associated gene expression was upregulated, and this effect was increased over time. Significant upregulation of apoptotic and neurotoxic genes, as well as genes associated with the JAK-STAT and NF-κB pathways was also observed over time. Deficiencies in many of these immune effectors, achieved via prion infection of knock-out mice, did not substantially influence symptom development or disease progression, suggesting their upregulation is another pathological effect, rather than causative or directly contributing [17].

The microglia response in prion disease is distinct from other neurodegenerative disorders at the RNA level, as revealed by RNA-seq analysis of prion-infected mice and appears to be associated with integrin CD11c/18 activation [18]. To further understand the role of microglia in prion disease progression, an inhibitor of the functional microglia marker CSF-1R (PLX5622) was utilized [17]. In accordance with their functional role of local immune cell, microglia responses to prions included neuroinflammation, which ultimately becomes pathological. When microglia are depleted, however, even after disease progression has begun, the prion infection was significantly more aggressive, as evidenced by both increased levels of PrP^Sc^ present and advanced progression of prion disease [17]. This suggests that while microglial responses contribute to prion disease via chronic neuroinflammation, this response still seems to serve a critical role in repressing or delaying prion inundation.

Given the contributions of the neuroinflammatory astrocyte phenotype identified in neurological conditions ranging from SCI to AD, the potential role of these reactive cells in prion diseases is of significant interest. Investigation into both prion disease mouse models and human disease samples confirmed the abundant presence of not only C3 + reactive astrocytes, but also C3 + PrP^Sc^ + astrocytes [38] (Fig. 1i). This result suggests that prion-propagating astrocytes adopt an A1-like phenotype, which may suggest a mechanism by which a diseased astrocyte undergo phenotypic polarization, a function that may apply to other pathologies. Interestingly, further analysis of these C3 + PrP^Sc^ + astrocytes revealed gene expression profiles were similar to, yet still distinct from, reported A1-like astrocytes [38]. RNA-seq analysis of prion-infected mice also confirmed a unique gene expression signature of astrocytes, with features from both neuroinflammatory and neuroprotective profiles, indicating a possible unique disease-associated variation within the A1-like phenotypic responses [18] (Fig. 1i).

As observed in other neurological conditions, A1-associated RNA markers were identified as upregulated in the human cortical tissue of sCJD samples, when compared to age-matched controls [126]. Expression patterns of two A1-associated markers, C3 and guanylate binding protein 2 (GBP2), both of which are known to colocalize with astrocytes in sCJD, were further examined between different patient samples to determine the relationship between neuroinflammatory reactive astrocyte protein expression and disease presentation [38, 126]. RT-qPCR analysis of prion-infected tissue found that the degree of C3 upregulation varied between different patient genotypes, specifically the presence of a methionine (M) or valine (V) at position 129 of the *PRNP* gene, with homozygosity being more common in sCJD. C3 upregulation was more significant in 129MM samples, compared to 129VV [126]. Given the function of C3 and other complement components, this may suggest that neuroinflammation is a significant contributor to disease in 129MM sCJD patients. It may also suggest that astrocyte-propagation of prion disease is a larger factor in 129MM sCJD disease, but more investigation is required. GBP2 upregulation was found to be significantly positively associated with disease duration [126]. While the implications of this are still unclear, it may indicate a greater role for activated microglia in sCJD, in which neuroprotective microglial functions over time give rise to A1-like astrocyte polarization [126]. Overall, these findings may suggest that the individual variations within the A1-like reactive phenotype are associated with disease progression in sCJD, and this may apply to other neurological diseases. Continued investigation into other A1-associated markers may provide further insight into the molecular basis of neuropathology variation and highlight treatment targets (Table 1).

To investigate the therapeutic potential of targeting the reactive astrocyte response in prion disease, IL1-α, TNF-α, and C1q triple knock out (TKO) mice were infected with prions [38]. Unexpectedly, the abolishment of A1-like polarization capacity was associated with microglia dysregulation and exacerbated disease progression [38]. This surprising finding, which is in direct contrast to those observed in many other neurodegenerative disorders, may hold implications for the underlying biological function of the neurotoxic reactive response. Similar paradoxical findings for the roles of neuroinflammatory C3 + neuroglia cells have been reported in retinitis pigmentosa, specifically C3 + microglia [111]. While detrimental at large scales, it may be possible that in local contexts or in the context of diseases that spread in an infection-like manner, the reactive astrocyte response ultimately serves a protective function [38]. While the prion disease will ultimately overcome such a response, in the early stages of the disease, A1-like reactive astrocytes may initially work cooperatively with microglia to suppress the spread of infection.

## Reactive astrocytes in infectious CNS disease

The potential role of A1-like astrocytes in infectious diseases of the CNS has received comparatively little attention to date.

### Bacterial

While very little has been reported on A1-like phenotypes in specific bacterial infections, there have been some investigations of general Gram-negative bacterial infections or endotoxemia, typically modelled using LPS stimulation. Cognitive impairment is a known effect of LPS exposure in the CNS, likely a consequence of neuroinflammation [134]. In a mouse model treated with high doses of LPS, changes in the concentrations of different cytokines were detected in CSF, specifically IL-1β [134]. This interleukin is a downstream effector of the activated NLRP3 inflammasome, and *Nlrp3* knockout mice treated with LPS were protected from cognitive impairment [134]. To understand the underlying cellular mechanisms, cultured primary mouse astrocytes were exposed to LPS-stimulated MCM [134]. As expected, qRT-PCR analysis revealed the upregulation of A1-associated markers. When microglia were exposed to nigericine, a NLRP3 inflammasome activator, in addition to LPS, the resulting MCM induced an A1-associated gene profile and increased C3 expression, compared to just LPS, in cultured astrocytes. Furthermore, when this experiment was repeated in astrocyte and neuron co-cultures, neurotoxicity significantly increased [134]. These results suggest bacterial toxin exposure in the CNS activates microglia, as well as the NLRP3 inflammasome, both of which appear to contribute to pro-inflammatory astrocyte polarization and associated neurotoxicity. Another cytokine of interest in bacterial infections with neurological symptoms, in this case for its proposed homeostatic functions, is interferon gamma (IFNγ) [80]. In a mouse model of endotoxemia and associated cognitive impairment, IFNγ levels increased in plasma and CSF, a finding that was consistent with the results collected from human patients with sepsis [80]. In the LPS-treated mice, IFNγ signaling was pronounced in the hippocampus and was associated with reduced neurogenesis and increased cognitive impairment [80]. Furthermore, in primary mixed-cell culture, IFNγ signaling was associated with microglia-facilitated A1-like polarization of astrocytes and neurotoxicity, specifically by increasing microglia release of TNFα and IL1-α. This effect was significantly reduced by inhibition of IFNγ signaling with a IFNγ receptor (IFNγR) knockout mouse model [80]. These findings provide promising evidence that the neurotoxic reactive astrocyte response contributes to CNS bacterial infection pathology.

### Parasitic

Neurotoxic astrocyte reactivity in the context of parasitic infections in the CNS is another area of research that has only recently begun to be investigated. One of the more well-recognized neuroparasitic infections is caused by the protozoa *Toxoplasma gondii,* infecting approximately one-third of the global population [51]. While *T. gondii* infection typically causes few to no symptoms in most people, severe effects can arise in fetuses and people who are immunosuppressed. Toxoplasmic encephalitis (TE) describes one such outcome, in which neuronal death occurs due to the *T. gondii* infection; however, the underlying mechanisms and roles of glial cells have not been fully elucidated [51]. An immunofluorescence assay of tissue collected from a mouse model of TE revealed C3 + -astrocytes and western immunoblot confirmed increased C3 protein expression in the TE model when compared to both control and chronic infection, suggesting A1-like polarization in severe *T. gondii* neuropathology [51]. In vitro analysis using primary mouse astrocytes exposed to *T. gondii* excreted-secreted antigens (TgESAs) resulted in polarization to an A1-like phenotype, independent of microglia, as evidenced by upregulated reactive astrocyte transcript expression and C3 levels [51] (Fig. 1j). This induction occurred independently of microglia and was dependent on the NF-κB pathway, as treatment with an NF-κB inhibitor attenuated A1-like polarization [51]. Another parasitic infection that can have neurological consequences is that caused by *Angiostrongylus cantonensis*. The resulting food-borne parasitic disease Angiostrongyliasis can cause symptoms ranging from fever and vomiting to meningitis and meningoencephalitis [147]. While the appropriate treatment can typically clear the infection, some debilitating symptoms can become chronic. In the CNS of an infected mouse model, *A. cantonensis* has been observed to induce demyelination with coinciding upregulation of IL-17A [147]. Treatment of mice with IL-17A neutralizing antibodies was associated with both attenuated corpus callosum demyelination and reduction in local A1-like astrocytes compared to untreated subjects [147]. Additional in vitro experiments in which astrocytes exposed to IL-17A adopted a C3 + A1-like phenotype suggest that IL-17A contributes to astrocyte polarization [147]. In addition, RNA-seq analysis and siRNA interference experiments identified the IL-17RASTAT3/SOCS3 pathway as an important mediator of the IL-17A response in the context of *A. cantonensis* infection, with IL17-A serving a regulatory role of astrocytic SOCS3 expression [147]. Altogether, recent evidence has shown that A1-like neurotoxic astrocytes contribute to neuroparasitic disease.

### Viral

Viral infections that infiltrate the CNS can cause debilitating chronic illness or have fatal outcomes, a topic that is extensively reviewed by Koyuncu et al. [64]. Given their roles in neuroinflammation, the contributions of microglia and astrocytes in such diseases is of importance. One study investigating reactive astrocyte phenotypes in a viral disease, canine distemper demyelinating leukoencephalitis, confirmed the increased presence of A1-like astrocytes in CNS tissue of infected samples, as indicated by gene expression analysis and staining for A1-markers SRGN and ACSL5 [60] (Table 1). This is consistent with the observation of A1-like astrocytes in the non-infectious human demyelinating disease MS. Immunohistochemical evaluation of distemper lesions revealed that the abundance of A1-like cells, as well as a progressive loss of astrocytic AQP4, was correlated with disease severity [60].

Several viral infections are not primarily localized to the CNS, but in some cases can infiltrate and go on to elicit neurological damage. Herpes simplex virus type 1 (HSV-1) is the cause of one such debilitating neuroviral disease, HSV-1 encephalitis (HSE). HSV-1 can infect neurons and astrocytes, and glial cell activation is a known feature of HSE [42]. To characterize the expression profiles of these activated glial cells in the context of HSE, mixed primary cortical cell cultures consisting of mouse neurons, oligodendrocytes, microglia, and astrocytes underwent HSV-1 exposure [42]. Astrocytes were preferentially infected by HSV-1, with the uninfected but local astrocytes becoming hypotrophic in response, while the infected astrocytes adopted a hypertrophic morphology [42]. Further, upregulation of TNF-α and CXCL10 was detected in HSV-1 infected primary cortical cells via qRT-PCR. While not definitive evidence of A1-like polarization, this finding is consistent with pro-inflammatory phenotypic changes; however, neuroprotective phenotype-associated Cox2 was also upregulated, suggesting a mixed response [42]. While further investigation is needed, these results may hold important implications for other chronic neuroviral infections, including HIV-associated neurocognitive disorders (HAND). Interestingly, in HAND several HIV-1 viral proteins, the expression of which is frequently associated with neuropathogenesis, have been shown to alter astrocyte function and induce activation, including Tat, Vpr, Nef, gp120, and gp41 [32, 46, 49, 84, 116, 118].

## Conclusion

Given their abundance in a variety of neuropathologies, further research is needed to explore a potential role for pathology-associated astrocytes in other neurological disorders and neuroinfectious diseases. Future investigations should attempt to utilize a broad array of molecular markers and functional phenotypes when describing reactive astrocyte polarization states, rather than adhering to strict A1/A2 classifications (Table 1). As the literature characterizing contributions of A1-like astrocytes to disease continues to expand, the question of potential therapeutic applications arises. Investigations into targeting this phenotype is becoming an area of increasing interest and may ultimately lead to the development of novel diagnostic evaluations or treatment plans. Targeting or blocking astrocyte polarization may prove to be an effective avenue of symptom management and treatment for a host of neurodegenerative or neuroinflammatory disorders. The selective serotonin reuptake inhibitor (SSRI) Fluoxetine, commonly prescribed for MDD, was also found to inhibit neurotoxic astrocyte polarization upon inflammatory stimulation both in vitro and in vivo. The increased concentration of A1-associated markers in a CMS mouse model was rescued with Fluoxetine treatment, which was associated with reduced depressive and anxiety behaviors. Using pharmacological inhibitors and siRNA technology, astrocytic 5HT_2B_R and downstream β-arrestin2 signaling were identified as the targets of the Fluoxetine-mediated inhibition of A1-like astrocyte polarization [31]. Recently, NLY01, a GLP-1R agonist, has been investigated as a neuroprotective agent in PD, and was found to directly prevent microglia from inducing astrocyte polarization [140]. These studies suggest that both current well-established therapies and those yet to be developed could be of use as neurotoxic reactive astrocyte inhibitors applicable to a wide array of neuropathologies. Continued investigation into the near-ubiquitous pathological roles of these reactive pro-inflammatory A1-like astrocytes will have important implications for how neuropathologies are studied and ultimately treated.

## Data Availability

Not applicable.

## Abbreviations

CNS: Central nervous system
BBB: Blood-brain barrier
AQP-4: Aquaporin-4
ROS: Reactive oxygen species
GFAP: Glial fibrillary acidic protein
LPS: Lipopolysaccharide
TLR4: Toll-like receptor 4
PAMPs: Pathogen-associated molecular patterns
DAMPs: Damage-associated molecular patterns
HMGB1: High-mobility group protein box-1
TNF-α: Tumor necrosis factor-α
IL-1α: Interleukin-1α
C1q: Complement component subunit 1q
C3: Complement component 3
MCM: Microglia-conditioned media
NO: Nitric oxide
Drp1: Dynamin-related protein 1
(Fis1) receptor: Mitochondrial fission 1
Aβ42: Amyloid-β 42
TGF-β: Transforming growth factor β
MFG‐E8: Milk fat globule epidermal growth factor 8
GM-CSF: Granulocyte-macrophage colony-stimulating factor
PK2: Prokineticin-2
PKR1: PK2 receptor 1
SCN: Suprachiasmatic nucleus
K_ir_4.1: Inwardly-rectifying K^+^ channel
mPFC: Medial prefrontal cortex
CSDS: Chronic social defeat stress
CMS: Chronic mild stress
HPA: Hypothalamic-pituitary-adrenal
POCD: Post-operative cognitive dysfunction
BDNF: Brain-derived neurotrophic factor
GDNF: Glial cell line-derived neurotrophic factor
PCD-1: Programmed cell death-1
STAT3: Signal transducer and activator of transcription 3
NF-κB: Nuclear transcription factor-κB
CSPG: Chondroitin sulfate proteoglycan
ERK1/2: Extracellular signal-regulated kinase 1/2
OLIG2: Oligodendrocyte transcription factor 2
SMAD: Smaand Mad-related protein
GSPT1: G1 to S phase transition 1
NOS: Nitric oxide synthase
GSI: γ-Secretase inhibitor
RGMa: Repulsive guidance molecule a
AD: Alzheimer’s disease
PD: Parkinson’s disease
HD: Huntington’s disease
ALS: Amyotrophic lateral sclerosis
MS: Multiple sclerosis
MHCII: Major histocompatibility complex II
IBA1: Ionized calcium-binding adaptor molecule 1
ALDH1L1: Aldehyde dehydrogenase 1 L1
C3aR: C3a receptor
TMT: Trimethyltin
ADE: Astrocyte-derived exosome
SR: Serine racemase
NMDAR: NMDA receptor
GLP-1R: Glucagon-like peptide-1 receptor
MN: Motor neuron
SOD1: Superoxide dismutase 1
RRMS: Relapsing remitting MS
EAE: Autoimmune encephalomyelitis
Cx43: Connexin 43
Cx47: Connexin 47
CAA: Cerebral amyloid angiopathy
FDD: Familial Danish dementia
Adan: Danish amyloid
BBS: Bardet-Biedl Syndrome
PSD95: Post-synaptic density 95 protein
LSD: Lysosomal storage diseases
NCL: Neuronal ceroid lipofuscinosis
INCL: Infantile neuronal ceroid lipofuscinosis
PPT1: Palmitoyl-protein thioesterase-1
PAT: Palmitoyl-acyl transferase
APT1: Acyl-protein thioesterase-1
PrP: Prion protein
UPR: Unfolded protein response
TSE: Transmissible spongiform encephalopathies
FFI: Fatal Familial Insomnia
GSS: Gerstmann-Sträussler-Scheinker disease
vCJD: Variant Creutzfeldt-Jakob Disease
sCJD: Sporadic CJD
BSE: Bovine spongiform encephalopathic
GBP2: Guanylate binding protein 2
M: Methionine
V: Valine
TKO: Triple knockout
IFNγ: Interferon gamma
IFNγR: IFNγ receptor
TE: Toxoplasmic encephalitis
TgESAs: *T. gondii* Excreted-secreted antigens ()
HSV-1: Herpes simplex virus type 1
HSE: HSV-1 encephalitis
HAND: HIV-associated neurocognitive disorders
